# Molecular mechanisms and pharmacological interventions in the replication cycle of human coronaviruses

**DOI:** 10.1590/1678-4685-GMB-2020-0212

**Published:** 2020-11-23

**Authors:** Fernando Moreira Simabuco, Rodrigo Esaki Tamura, Isadora Carolina Betim Pavan, Mirian Galliote Morale, Armando Morais Ventura

**Affiliations:** 1 Universidade Estadual de Campinas, Faculdade de Ciências Aplicadas (FCA), Laboratório Multidisciplinar em Alimentos e Saúde (LABMAS), Limeira, SP, Brazil.; 2 Universidade Federal de São Paulo (UNIFESP), Departmento de Ciências Biológicas, Diadema, SP, Brazil.; 3 Universidade Estadual de Campinas, Faculdade de Ciências Farmacêuticas (FCF), Campinas, SP, Brazil.; 4 Universidade de São Paulo (USP), Departamento de Radiologia e Oncologia, Faculdade de Medicina, Centro de Oncologia Translacional, Instituto do Câncer do Estado de São Paulo (ICESP), São Paulo, SP, Brazil.; 5 Universidade de São Paulo (USP), Instituto de Ciências Biomédicas (ICB), Departamento de Microbiologia, São Paulo, SP, Brazil.

**Keywords:** Coronavirus, SARS-CoV-2, COVID-19, viral replication, RNA virus

## Abstract

SARS-CoV-2 (Severe Acute Respiratory Syndrome Coronavirus 2), as well as SARS-CoV from 2003 along with MERS-CoV from 2012, is a member of the Betacoronavirus genus of the Nidovirales order and is currently the cause of the pandemic called COVID-19 (or Coronavirus disease 2019). COVID-19, which is characterized by cough, fever, fatigue, and severe cases of pneumonia, has affected more than 23 million people worldwide until August 25^th^, 2020. Here, we present a review of the cellular mechanisms associated with human coronavirus replication, including the unique molecular events related to the replication transcription complex (RTC) of coronaviruses. We also present information regarding the interactions between each viral protein and cellular proteins associated to known host-pathogen implications for the coronavirus biology. Finally, a specific topic addresses the current attempts for pharmacological interventions against COVID-19, highlighting the possible effects of each drug on the molecular events of viral replication. This review intends to aid future studies for a better understanding of the SARS-CoV-2 replication cycle and the development of pharmacological approaches targeting COVID-19.

## Introduction

In 2002 and 2003, the first coronavirus outbreak started in Guangdong province in China, causing a total of 8,098 cases and 774 deaths worldwide, with a mortality rate of roughly 9% (Fehr and Perlman, 2015). This outbreak caused Severe Acute Respiratory Syndrome, or SARS. In 2012, another outbreak started in the middle-east, called MERS (Middle East Respiratory Syndrome), with an initial mortality rate of nearly 50%, which was controlled in the ensuing years. In 2014, a total of 855 cases and 333 deaths were reported by the MERS-CoV, with a 40% mortality rate, according to the European Center for Disease Prevention and Control (Fehr and Perlman, 2015). Before these two outbreaks, coronaviruses were believed to cause only self-limiting mild respiratory tract infections in humans.

In December 2019, a group of patients from the city of Wuhan, Hubei province, China, was initially diagnosed with a pneumonia of unknown etiology. The cases were epidemiologically linked to a seafood and wildlife market within the city (Rothan and Byrareddy, 2020). Afterward, reports predicted the appearance of a potential coronavirus outbreak since the reproduction number for the new 2019 coronavirus disease (COVID-19, as named by the WHO on February 11th 2020) was significantly greater than 1, estimated between intervals of 2.24 to 3.58 (Zhao *et al.*, 2020). Since then, until August 25th, 2020, the number of infected people has reached 23,677,221 cases and 813,802 deaths worldwide (Coronavirus Resource Center, John Hopkins University).

Symptoms of COVID-19 infection appear after an incubation period of approximately 5.2 days. The period between the onset of COVID-19 symptoms and death has a median of 14 days, varying from 6 to 41 days. The most common symptoms at the start of COVID-19 disease are cough, fever, and fatigue, and some cases may involve sputum production, headache, hemoptysis, diarrhea, dyspnea, and lymphopenia (Rothan and Byrareddy, 2020).

## Taxonomy and genomic organization of coronaviruses

The Coronaviridae family is part of the Nidovirales order and it can be divided into two subfamilies: Coronavirinae and Torovirinae. The Coronavirinae subfamily has four genera: alpha-, beta-, gamma-, and delta-coronaviruses. SARS-CoV-2 (also called 2019-nCoV) is the coronavirus that causes COVID-19 and, together with SARS-CoV and MERS-CoV, are part of the Betacoronavirus genus. A study showed that SARS-CoV-2 possesses greater similarity (88% identity) to two SARS-like coronaviruses derived from bats (bat-SL-CoVZC45 and bat-SL-CoVZXC21), collected in 2018 in Zhoushan, eastern China, than with SARS-CoV (about 79%), and MERS-CoV (about 50%) (Lu R *et al.*, 2020). Another study has shown that the SARS-CoV-2 genome is 91.02% similar to Pangolin-CoV, despite a higher identity of 96.2% between SARS-CoV-2 and another bat coronavirus (RaTG13) (Zhang T *et al.*, 2020). This suggests that SARS-CoV-2 has originated from bats and might have pangolins as an intermediate host species, since five key amino acid residues (LFQNY) in the Receptor-Binding Domain (RBD) of S protein, involved in the interaction with human ACE2 receptor, are similar among Pangolin-CoV and SARS-CoV-2, but not with RaTG13 (Zhang T *et al.*, 2020).

**Figure 1:**
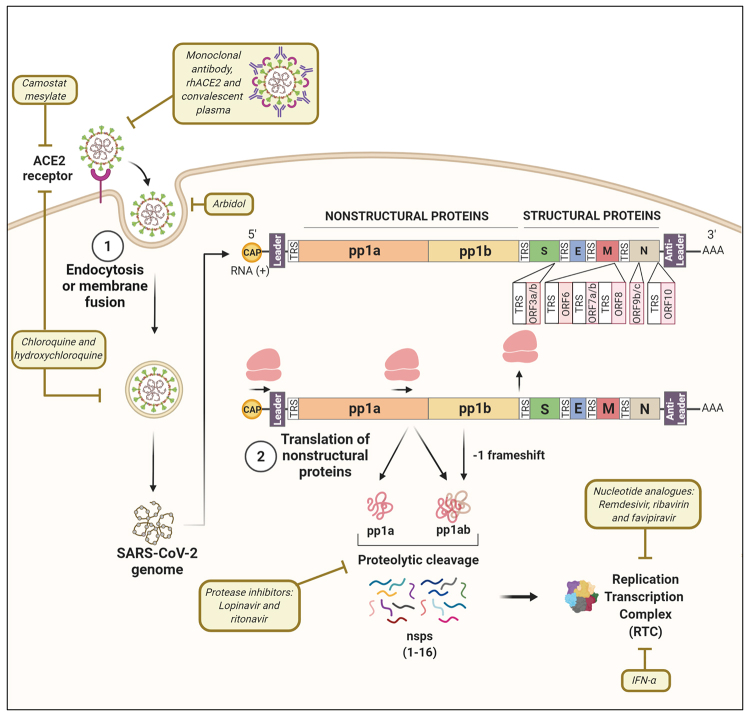
Molecular mechanisms related to the production of non-structural proteins (nsps) and assembly of the SARS-CoV-2 replication and transcription complex (RTC). Process 1: after recognition of the ACE2 (Angiotensin Converting Enzyme 2) cell receptor, the viral nucleocapsid is released into the cytoplasm by endocytosis, or fusion of the viral envelope, with the cell membrane. Process 2: the translation of the pp1a and pp1b genes from the 5’-capped and 3’-polyadenylated genome (+) of the virus produces the pp1a or pp1ab polyproteins, the latter being generated by a -1 frameshift of ribosomes. These polyproteins are then cleaved by viral proteases generating 16 virus nonstructural proteins (nsps), some of which are used to assemble the RTC, including the RNA-dependent RNA polymerase (RdRp or nsp12). Pharmacological interventions targeting specific points of the replication cycle of coronaviruses are highlighted. RTC: Replication and transcription complex; RdRp: RNA-dependent RNA polymerase.

The coronavirus genome is composed of a single-stranded positive RNA (ssRNA+), therefore included in class IV of the Baltimore classification (Baltimore, 1971). The genomic organization of coronaviruses can be divided into two main parts, the genes encoding the non-structural poly-proteins pp1a and pp1b and the genes encoding the structural genes, including the S, E, M and N genes, as shown in Figure 1.

## The replication and the non-structural proteins (nsps) of coronaviruses

The replication of SARS-CoV-2 begins with the translation of the pp1a and pp1b polyproteins from the single-stranded positive polarity genomic RNA (ssRNA+), which is 5’-capped and 3’-polyadenylated (Figure 1). The pp1b polyprotein is produced in fusion with pp1a through a -1 frameshift mechanism, generating 2 polyproteins called pp1a (without frameshift) and pp1ab (with frameshift) (Brierley *et al.*, 1989). The frameshift occurs because of a slippery sequence in the genome (5’-UUUAAAC-3’) and a pseudoknot structure in the secondary structure of the RNA before the STOP codon of pp1a ORF, causing a pause in the ribosome reading and the translation of pp1b ORF in fusion with pp1a ORF, thus generating pp1ab (Fehr and Perlman, 2015).

Once produced, the pp1a and pp1ab polyproteins undergo proteolytic cleavage, forming a total of 16 proteins, detailed in Table 1 and outlined in Figure 1. The cleavage is generated by 2 proteases: nsp3, which is a Papain-like protease (PL^pro^) and nsp5, which is a 3C-like protease (3CL^pro^). Nsp3 cleaves the sites between nsp2 to nsp4, therefore generating nsp1, nsp2, and nsp3. On the other hand, nsp5 cleaves the other sites, generating the other non-structural proteins (Ziebuhr *et al.*, 2000; Báez-Santos *et al.*, 2015).

**Table 1:** Non-structural proteins of coronaviruses and their functions.

Non-structural proteins (nsps)	Functions	References
nsp1	Promotes cell mRNA degradation and blockage of host cell translation and innate immune response	(Huang *et al.*, 2011; Tanaka *et al.*, 2012)
nsp2	Unknown function, binds to prohibitins	(Cornillez-Ty *et al.*, 2009)
nsp3	Papain-like protease (PL^pro^), cleaves the viral polyproteins and blocks the innate immune response, has multiple domains	(Lei *et al.*, 2018)
nsp4	Transmembrane scaffold protein, formation of DMVs (Double Membrane Vesicles)	(Gadlage *et al*., 2010)
nsp5	3C-like protease (3CL^pro^), cleaves viral polyproteins, inhibits IFN signaling by cleaving STAT2	(Stobart *et al.*, 2013; Zhu *et al.*, 2017)
nsp6	Transmembrane scaffold protein, formation of DMVs (Double Membrane Vesicles), inhibits autophagosome	(Angelini *et al.,* 2013; Cottam *et al.*, 2014)
nsp7	Forms a hexadecameric complex with nsp8	(te Velthuis *et al.*, 2012)
nsp8	Forms a hexadecameric complex with nsp7, can act as primase	(te Velthuis *et al.*, 2012)
nsp9	Dimerization and RNA binding	(Zeng *et al.*, 2018)
nsp10	Cofactor for nsp14 and nsp16	(Decroly *et al.*, 2011)
nsp11	In pp1a, it consists of a small peptide with unknown function. In pp1ab polyprotein, nsp11 is translated into nsp12 due to the -1 frameshift between pp1a and pp1b	(Fehr and Perlman, 2015)
nsp12	RNA-dependent RNA polymerase (RdRp)	(te Velthuis *et al.*, 2010)
nsp13	RNA helicase, 5’ triphosphatase	(Jia *et al.*, 2019)
nsp14	Exo-ribonuclease 3’-5’ proofreading, N7-methyltransferase	(Chen Y *et al.*, 2009; Bouvet *et al.*, 2012)
nsp15	Endo-ribonuclease, evasion of apoptosis and dsRNA cell sensors	(Bhardwaj *et al.*, 2006; Deng *et al.*, 2017)
nsp16	2’-O-methyltransferase; inhibits RIG-I and MDA5, negatively regulating innate immunity	(Decroly *et al.*, 2011; Shi *et al.*, 2019)

**Figure 2:**
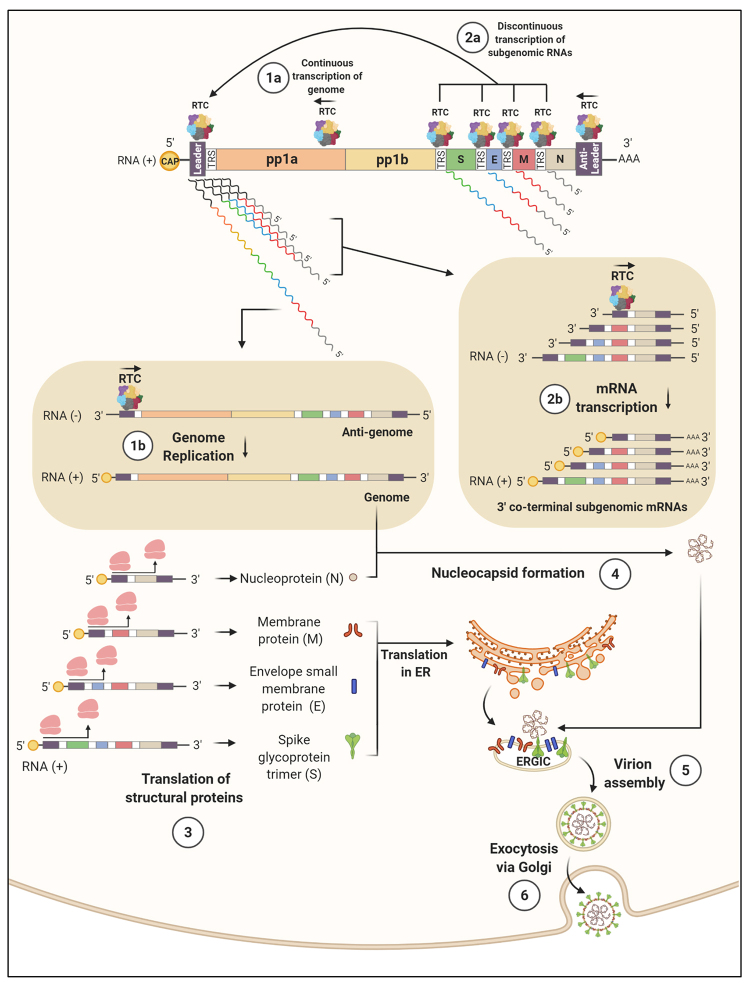
Molecular events related to the expression of structural proteins, replication of the genome, and assembly of the SARS-CoV-2. Processes 1a and 1b: the synthesis of RNA (-) by RTC, initiated in the 3’ anti-leader sequence of the genome (+), may occur continuously, generating a complete copy of the genome called antigenome (-). The antigenome is then used by RTC to produce multiple copies of the genome (+). Processes 2a and 2b: RNA synthesis by RTC may, however, be temporarily interrupted when a TRS is copied. The newly synthesized RNA (-) is then transferred to the 5’ end of the genome, where the complementarity of sequences allows the RNA (-) synthesis to continue in the leader TRS, merging the sequences between body and leader TRSs. In turn, these subgenomic chimeric RNAs (-) serve as templates for the continuous synthesis of subgenomic mRNAs (+). Process 3: the structural S, E, M, and N proteins are then translated from the 3’-co-lateral subgenomic mRNAs (+), where S, E, and M proteins are produced in the rough endoplasmic reticulum. Process 4: the N protein produced in the cytosol interacts with the viral genome (+), forming the nucleocapsid. Process 5: membrane proteins S, M, and E then interact with viral nucleocapsids to form virions in the ERGIC. Process 6: finally, the virions are externalized from the cell by exocytosis via the Golgi pathway. TRS: Transcriptional Regulatory Sequences; ER: endoplasmic reticulum; RTC: Replication Transcription Complex. ERGIC: Endoplasmic Reticulum - Golgi Intermediate Compartment.

The next processes of coronavirus replication and transcription are outlined in Figure 2 below. Once produced and processed, part of the non-structural proteins along with nsp12, the RNA-dependent RNA polymerase (RdRp), assemble the Replication and Transcription Complex (RTC). RTC acts primarily by producing a set of single-stranded negative RNAs (ssRNA-), including copies of the genomic RNA and subgenomic RNAs, which will then serve as templates for the production of the genome and mRNA, respectively. These intermediate negative RNA molecules are about 1% as abundant as their respective positive counterparts and contain anti-leader sequences, present in the 5’ untranslated region (UTR) of the antigenome and in the 3’-UTR of the genome (Sethna *et al.*, 1991). On the other hand, in the 5’-UTR of the viral genome and 3'-UTR of the antigenome there are leader sequences. The leader and anti-leader sequences are used by the RTC to initiate replication and transcription. In the 5’-UTR of the genome and at the beginning of each ORF of the structural genes, there are other regulatory regions called Transcriptional Regulatory Sequences (TRS), as shown in Figures 1 and 2. During the transcription of ssRNA molecules, two mechanisms may happen, according to the currently established model of replication of coronaviruses (Pasternak *et al.*, 2006; Sawicki *et al.*, 2007):

The RTC, after binding to the 3’ anti-leader sequence of the viral genome, initiates the synthesis of negative RNA throughout the molecule until it finds the leader region in the 5’ end, generating a complete copy of negative polarity of the genome, called antigenome. This antigenome will serve as template for the synthesis of the genome (+) (Figure 2, processes 1a and 1b).

2) The RTC may, however, temporarily pause the transcription in each of the TRS regions of each ORF and continue in the 5’-UTR of the genome, given the complementarity of the TRS regions. Therefore, a leader region is incorporated into each RNA, generating subgenomic RNAs of negative polarity. These subgenomic RNAs will serve as a templates for the mRNA (+) synthesis, containing their 3’ regions co-terminal to the genomic RNA (Figure 2, processes 2a and 2b). This process is often called “copy-choice” mechanism and is manifested in other viruses, enabling recombination, as will later be discussed (Simon-Loriere and Holmes, 2011).

Once the complete genomic RNA and mRNAs of each structural ORFs are produced, the translation of these genes begins (Figure 2, process 3), generating the S, E, M and N proteins, which are essential for the assembly of the viral particles, among others (ORFs 3a, 6, 7a / b, 8 and 9). The specific functions of these proteins will be described later. The viral replication cycle then continues through the interaction of the N protein with the viral genomic RNA, forming the nucleocapsid in the cytosol (Figure 2, process 4). On the other hand, the production of S, E, and M proteins are directed to the rough endoplasmic reticulum (RER) (Fehr and Perlman, 2015). Finally, interactions mediated between these structural proteins culminate in the recruitment of nucleocapsids into the compartment between the RER and the Golgi apparatus called ERGIC (Endoplasmic Reticulum - Golgi Intermediate Compartment) and finally in the exocytosis of the viral particles (Figure 2, processes 5 and 6) (Fehr and Perlman, 2015).

An important feature related to the replication of coronaviruses is the high rates of mutation and recombination, which alters viral protein properties, host range, and pathogenicity. For example, there are reports of heterologous recombination between subgroup A Betacoronavirus and other viruses, since some of these coronaviruses have the hemagglutinin esterase gene, derived from the Influenza C virus (Zeng *et al.*, 2008). The recombination between coronaviruses targeting different species is also largely reported in the family and may explain the similarity between the genome sequence of human SARS-CoV-2 and bat and pangolin coronaviruses, as already discussed (Lau *et al.*, 2020; Lu R *et al.*, 2020; Zhang T *et al.*, 2020). Three aspects may explain this increased capacity for recombination/mutation:

1) the RdRp of coronaviruses has low fidelity. Although a 3’-5’ exonuclease proofreading activity is reported, the mutation rate of this polymerase is about 2.0 × 10^-6^ mutations per site, per replication cycle (Eckerle *et al.*, 2010);

2) the unique RNA replication mechanism using the TRS motifs, known as the “copy-choice” mechanism, may induce homologous RNA recombination between genes of different coronaviruses (Simon-Loriere and Holmes, 2011);

3) Coronaviruses have the largest genome (26-32 kb) among RNA viruses (Terada *et al.*, 2014).

Several studies have shown the modulation of cellular pathways by coronavirus proteins, favoring the viral cycle or impacting the viral pathogenesis, which is summarized in Table 2. Among these studies, interactions were found between the coronavirus nsp1 protein with the cyclophilins PPIA, PPIG, PPIH, and FKBP1A, FKBP1B that are capable of modulating the Calcineurin/NFAT pathway, which plays an important role in the activation of immune cells (Pfefferle *et al.*, 2011). The same study, which used the yeast two-hybrid system to demonstrate those interactions, showed that the inhibition of cyclophilins by cyclosporine A (CspA) blocked the replication of different CoVs, including the human coronaviruses SARS-CoV, CoV-229E and -NL-63; the feline CoV; and the avian infectious bronchitis virus (IBV). Another study has demonstrated the interaction between the SARS-CoV nsp2 and cellular prohibitins, suggesting that this nsp may be involved in the disruption of intracellular host signaling (Cornillez-Ty *et al.*, 2009).

**Table 2:** Interactions between coronavirus proteins and cellular proteins and/or pathways.

Viral protein	Interactions		References
	Viral	Cellular	
Nsp1		Cyclophilin (PPIA, PPIB, PPIH, PPIG, FKBP1A, FKBP1B)	(Pfefferle *et al.*, 2011)
Nsp2		Prohibitin	(Cornillez-Ty *et al.*, 2009)
Nsp3	N protein	TGF-β1 (indirect); STING-TRAF3-TBK1; RCHY1, p53 and IRF3	(Hurst *et al.*, 2013; Chen *et al.*, 2014; Ma-Lauer *et al.*, 2016; Li *et al.*, 2016)
Nsp5		STAT2	(Zhu *et al.*, 2017)
Nsp6		Autophagosome	(Cottam *et al.*, 2014)
Nsp7	Nsp8		(te Velthuis *et al.*, 2012)
Nsp8	Nsp7		(te Velthuis *et al.*, 2012)
Nsp9		TRIM59 and MIB1	(Gordon *et al.*, 2020)
Nsp10	Nsp14 and Nsp16		(Decroly *et al.*, 2011)
Nsp13		TBK1, TBKBP1, TLE1, 3, and 5	(Gordon *et al.*, 2020)
Nsp15		Apoptosis and dsRNA cell sensors; Rb	(Bhardwaj *et al.*, 2012; Deng *et al.*, 2017)
Nsp16		RIG-I and MDA5 (innate immunity)	(Shi *et al.*, 2019)
RTC		Translation initiation factors (eIF3E, eIF3F and eIF3I); Intracellular transport (SNARE proteins; SRP54a and SRP68 proteins); autophagy-related factors and ubiquitin-dependent ERAD components	(V’kovski *et al.*, 2019)
S	M	ACE2; TMPRSS2; apoptosis	(Yeung *et al.*, 2008; Neuman *et al.*, 2011; Yan *et al.*, 2020)
E	M protein	PALS1 (tight junction); BRD2 and BRD4; palmitoylations	(Boscarino *et al.*, 2008; Chen *et al.*, 2009; Teoh *et al.*, 2010; Gordon *et al.*, 2020)
M	E protein; N protein; S protein		(Chen *et al.*, 2009; Hurst *et al.*, 2009; Neuman *et al.*, 2011)
N	M protein; Nsp3	RNA interference machinery; NCL; NPM; NONO; PABP; HNRNPs; ribosomal proteins; caprin-1; G3BPs; GSK3; PACT; TRIM25; cyclin D; LARP1; CK2; UPF1; MOV10	(Chen *et al.*, 2002; Surjit *et al.*, 2006; Hurst *et al.*, 2009; Emmott *et al.*, 2013; Hurst *et al.*, 2013; Cui *et al.*, 2015; Ding *et al.*, 2017; Gordon *et al.*, 2020)
ORF3a		TRAF3 and ASC; caveolin-1; eIF2α and PERK	(Padhan *et al.*, 2007; Minakshi *et al.*, 2009; Siu *et al.*, 2019)
ORF6	Nsp8	karyopherin alpha 2 and karyopherin beta 1; NUP98-RAE1	(Kumar *et al.*, 2007; Frieman *et al.*, 2007; Gordon *et al.*, 2020)
ORF7a	ORF3	Type I IFN response; BST-2; cyclin D3/pRb pathway	(Yuan *et al.*, 2006; Dedeurwaerder *et al.*, 2014; Taylor *et al.*, 2015)
ORF10		Cullin 2 (CUL2) RING E3 ligase complex; ZYG11B	(Gordon *et al.*, 2020)

The PL^pro^ protein (nsp3) from SARS-CoV significantly triggered the activation of the TGF-β1 promoter through ROS/p38-MAPK/STAT3, correlating with the positive regulation of pro-fibrotic responses *in vitro* and *in vivo* (Li *et al.*, 2016). Another study showed that p53 downregulates SARS-CoV replication and is a target of nsp3 via an E3 ubiquitin ligase (Ma-Lauer *et al.*, 2016). The other viral protease, 3CL^pro^ (nsp5), cleaves STAT2, but not JAK1, TYK2, STAT1, and IRF9, which are key molecules of the JAK-STAT pathway, antagonizing the type I interferon signaling (Zhu *et al.*, 2017).

Coronavirus nsp6 is known to interfere with autophagy, limiting the autophagosomes diameter at the point of omegasome formation, which may favor viral infection by compromising the ability of the autophagy system to degrade viral components via lysosomes (Cottam *et al.*, 2014). Nsp9 seems to bind to E3 ubiquitin ligases TRIM59 and MIB1, which regulate antiviral innate immunity, and nsp13 may modulate the IFN pathway through TBK1 and TBKBP1 and the NF-κB pathway by TLE-1, -3, and -5 (Gordon *et al.*, 2020). Another study reported that the coronavirus endoribonuclease nsp15 is required for evasion of dsRNA sensors and apoptosis since the loss of nsp15 activity is related to the stimulation of a protective immune response, which attenuated the disease in mice (Deng *et al.*, 2017). Nsp16 can downregulate the activities of RIG-I and MDA5, inhibiting innate immunity to promote viral proliferation (Shi *et al.*, 2019).

Finally, an important study recently showed cellular pathways related to the coronavirus RTC, using MHV (mouse hepatitis virus) as a model (V’kovski *et al.*, 2019). The study identified that RTC interacts with translation initiation factors, which may not persist throughout the replication cycle but may be of transitory importance during specific phases of the replication cycle. In addition, the knockdown of some of these factors, such as eIF3E, eIF3F, and eIF3I, impacted the MHV replication. Proteins related to transport and intracellular organization were also related to RTC in this study.

## The structural proteins of coronaviruses

### The S protein

Among all the structural proteins of coronaviruses, the S, E, M, and N proteins are considered essential and their functions are described below. Homotrimers of the S protein form the spikes in the viral surface, which give the virion a crown or corona aspect and the name of the family Coronaviridae, this being responsible for binding to host receptors (Beniac *et al.*, 2006). The S protein has roughly 150 kDa and contains an N-terminal signal sequence that gives access to the endoplasmic reticulum (RER) for its synthesis, being strongly N-terminal glycosylated. The S protein is trimeric and a class I fusion protein, mediating host receptor binding (Bosch *et al.*, 2003). A recent study showed that the angiotensin-converting enzyme 2 (ACE2) is the cell receptor for SARS-CoV-2, as well as for SARS-CoV, trimeric protein S being its ligand (Yan *et al.*, 2020). In addition, the cellular serine protease TMPRSS2, targeting ACE2, facilitates the cellular entry of SARS-CoV and SARS-CoV-2 and a TMPRSS2 inhibitor, camostat mesylate, partially inhibits *in vitro* SARS-CoV-2 infection (Hoffmann *et al.*, 2020). Apart from this, coronavirus entry in cells requires S protein priming by cellular proteases, which includes TMPRSS2 action (Hoffmann *et al.*, 2020).

### The E protein

The E protein plays a role in the assembly and release of virions from cells, being involved in viral pathogenesis (DeDiego *et al.*, 2007). It is a small protein (~8-12 kDa) and is found in small amounts within the virion. Protein E is a homopentameric transmembrane protein, possessing an N-terminal ectodomain, a C-terminal endodomain, and ion channel activity, and such activity in SARS-CoV is not necessary for viral replication but impacts the viral pathogenesis (Nieto-Torres *et al.*, 2014). Recombinant viruses without protein E are viable but attenuated, unlike other structural proteins, although this effect is dependent on the virus type (DeDiego *et al.*, 2007). It interacts with the M membrane protein in the budding compartment of the cell, located in the ERGIC (Chen SC *et al.*, 2009). The SARS-CoV E protein interacts with the PALS1 protein (Protein Associated with Lin-Seven 1) via the PDZ binding motif in its C-terminal domain and delays the formation of tight junctions, altering epithelial morphogenesis (Teoh *et al.*, 2010). Another study showed that palmitoylation of the E protein is crucial for the assembly of murine coronavirus (Boscarino *et al.*, 2008). A recent study has shown that the SARS-CoV-2 E protein binds to BRD2 and BRD4, members of the bromodomain and extra-terminal domain (BET) family, which are known epigenetic readers (Gordon *et al*., 2020). These bromodomain proteins bind to acetylated histones and may regulate transcriptional processes, as well as viral proteins that interact with them, as demonstrated for the Influenza NS1 protein (Marazzi *et al.*, 2012).

### The M protein

The M protein presents three transmembrane domains and is responsible for the shape of virions, promoting the membrane curvature and binding to the nucleocapsid (Neuman *et al.*, 2011). It is the most abundant structural protein in the virion, with approximately 25-30 kDa and presenting a small glycosylated ectodomain at the N-terminal and a larger C-terminal endodomain that extends from 6 to 8 nm in the viral particle (Nal *et al.*, 2005). The M protein forms a dimer in the virion and can adopt two different conformations, interacting with the nucleocapsid N and S proteins (Neuman *et al.*, 2011). The interaction with the nucleocapsid N protein promotes the complete assembly of the virion and was mapped to occur between the C-terminal of the M protein endodomain and the N protein CTD (Hurst *et al.*, 2005). As already reported, its interaction with protein E has also been demonstrated (Chen *et al.*, 2009). The M protein directs most of the protein-protein interactions necessary for the assembly of coronaviruses. A study showed that the surface proteins of coronavirus S, M, and E present differential subcellular locations when expressed alone, suggesting that additional cellular or viral factors may be necessary for coordinated traffic to the viral assembly site in the ERGIC (Nal *et al.*, 2005).

Another study showed that the expression of the M protein alone is insufficient for the formation of virus-like particles (VLPs) (Bos *et al.*, 1996). However, when the M protein was expressed along with the E protein, the formation of VLPs occurred, suggesting the important role of the two proteins in producing the envelopes of coronaviruses. The formation of VLPs is an important tool for therapeutical purposes since many of them can be used for testing and producing vaccines. A recent study reported the use of the SARS-CoV-2 S protein, a truncated S protein, or VLPs containing the S, M, and E proteins as candidates for vaccines against COVID-19. All formulations presented effectiveness in animal experimentation, but only VLPs induced both humoral and T cell immune responses (Lu J *et al.*, 2020).

### The N protein

The N protein is the only viral nucleocapsid protein and it contains two domains. The two structural domains of the N protein, the N-terminal RNA binding domain (RBD; residues 45-181) and the C-terminal dimerization domain (DD; residues 248-365), do not interact with each other and are surrounded by flexible linkers (Chang *et al.*, 2006). It is reported that the N protein can bind to nsp3 to assist the binding of the viral genome to the RTC and the packaging of the encapsidated genome in virions (Hurst *et al.*, 2009; Chen Y et al., 2020). The interaction between N and nsp3 supports a model in which this interaction tethers the genome to newly translated RTCs at an early stage of infection (Hurst *et al.*, 2013). Also, one of the N protein domains is critical for the recognition of the M protein during virus assembly in cells. The interaction of the N protein and viral nucleocapsid with the membrane proteins S, E, and M for viral packaging takes place in the ERGIC, forming the mature virions that are then extruded from the cells by exocytosis via Golgi (de Haan and Rottier, 2005). The expression of the N protein increases the formation of VLPs, suggesting that the fusion of encapsidated genomes in the ERGIC improves viral envelope formation (Siu *et al.*, 2008). A recent study suggests that the SARS-CoV-2 N protein has properties, such as RNA-binding, oligomerization, and multiple low-complexity regions, which indicates its involvement in cellular stress granules and ribonucleoprotein condensates that may be important for viral genome replication and packaging (Cascarina and Ross, 2020).

Regarding cellular interactions, several targets of the N protein have been proposed. A study has shown that coronavirus the N protein is a viral suppressor of RNA silencing (VSR) since the ectopic expression of the SARS-CoV N protein could promote MHV-A59 coronavirus replication in RNAi-active cells but not in cells depleted for the RNAi machinery (Cui *et al.*, 2015). Apart from this, a proteomics study has demonstrated interactions between the coronavirus N protein and several cellular components, including ribosomal proteins, translation initiation factors, nucleolar proteins, helicases, and hnRNPs (Emmott *et al.*, 2013). Some of those interactions, such as NONO and poly(A)-binding protein (PABP), were potentially mediated by RNA and the interactions with caprin-1, G3BP-1, and G3BP-2, which are involved in the formation of cytoplasmic stress granules, explain the localization of the N protein in these cell structures. The interaction between the N protein and NCL is a possible explanation of how coronavirus N proteins can localize to the nucleolus of cells (Chen *et al.*, 2002). Finally, the impact of some cellular targets for viral replication was evaluated by RNA-interference depletion, demonstrating the functional importance of NCL, RPL19, or GSK3 proteins in the biology of coronavirus (Emmott *et al.*, 2013).

Another proteomic study has confirmed that the SARS-CoV-2 N protein binds to stress granule proteins, including G3BP-1and -2. Also, the study found interactions between the N protein and other host mRNA binding proteins, including the translational repressor LARP1 (regulated by mTOR), the protein kinases CK2, and the mRNA decay factors UPF1 and MOV10 (Gordon *et al.*, 2020). Other coronaviruses, including MERS-CoV and MHV, have been implicated in the modulation of stress granules formation (Raaben *et al.*, 2007; Nakagawa *et al.*, 2018). The inhibition of stress granules by MERS-CoV ORF4a favors its replication, thus suggesting that stress granule formation may be an antiviral response with possible therapeutic applications.

## Other ORFs of coronaviruses

Aside from the pp1a, pp1b, S, E, M, and N proteins, SARS-CoV-2 presents 9 more ORFs, called ORF3a, ORF3b, ORF6, ORF7a, ORF7b, ORF8, ORF9b, ORF9c and ORF10, in which much less information is known regarding their molecular mechanisms of action in the viral replication cycle. SARS-CoV ORF3a is reported to bind to TRAF3 and ASC, promoting TRAF3 ubiquitination and activation of the NLRP3 inflammasome (Siu *et al.*, 2019). Other studies have demonstrated that ORF3a presents binding affinities for caveolin-1 and calcium (Padhan *et al.*, 2007; Minakshi *et al.*, 2014). SARS-CoV ORF6 is localized in the ER and Golgi membranes in infected cells, binding to karyopherin alpha 2 and karyopherin beta 1 proteins and hindering STAT1 nuclear import and its function (Frieman *et al.*, 2007). SARS-CoV-2 ORF6 has been associated to the NUP98 and RAE1 proteins, which constitutes an interferon-inducible mRNA nuclear export complex that is degraded by other viruses, such as Influenza, to favor their replication (Satterly *et al.*, 2007; Gordon *et al.*, 2020). Another study showed that nsp8 interacts with ORF6, suggesting that the ORF6 protein plays a role in virus replication (Kumar *et al.*, 2007).

Coronavirus ORF7a has been recognized as a type I IFN antagonist only when in the presence of the ORF3 protein, protecting the virus from the antiviral state induced by this cytokine (Dedeurwaerder *et al.*, 2014). The ORF7a protein also binds to BST-2 (Bone marrow stromal antigen 2 or tetherin), an antiviral protein that restricts SARS-CoV infection, blocking its glycosylation, whereas the loss of ORF7a leads to a much greater restriction (Taylor *et al.*, 2015). The ORF7a expression has also been associated with cell cycle arrest at the G0/G1 phase in HEK 293 cells via the cyclin D3/pRb pathway (Yuan *et al.*, 2006). Another study has found that the translation of SARS-CoV ORF7b may be mediated by leaky scanning of ribosomes and that it localizes in the Golgi compartment and is incorporated into viral particles (Schaecher *et al.*, 2007).

A study has demonstrated that SARS-CoV ORF8 may have originated through recombination from SARS-related coronavirus from bats, which may have an impact on animal-to-human transmission (Lau *et al.*, 2015). Regarding the exclusive SARS-CoV-2 ORF10, this protein has been associated with the Cullin 2 RING E3 ligase complex (CUL2), by interaction with the ZYG11B protein, suggesting that ORF10 hijacks the CUL2 complex for ubiquitination and degradation, or the opposite (Gordon *et al.*, 2020).

## Analysis of cellular pathways related to coronavirus replication

To further analyze the cellular proteins related to coronavirus biology, we performed Ingenuity Pathway Analysis, using the list of proteins presented in Table 2. This enabled the generation of a canonical pathways list, shown in Table 3, that may be important or modulated during the replication cycle of coronaviruses. The top 10 pathways were selected based on their associated p-value and are also presented in detail in the Figures S1-S10. The modulation of key molecular players, such as p53 and mTOR pathways, the inhibition of the host immune response by restraint of IFN induction and changes in cell cycle and cell growth, have been highly associated with the coronavirus proteins. These events may create a proliferative state that favors viral replication and inhibits apoptosis, facilitating viral cycle progression.

**Table 3:** Ingenuity Pathway Analysis (IPA) reveals the top 10 canonical pathways related to the cellular proteins that interact with coronavirus proteins, as summarized in Table 2.

Ingenuity Canonical Pathways	-log (p-value)	Ratio (strength of association)*	Genes/Proteins (total number)
Role of PKR in Interferon Induction and Antiviral Response	8,84E00	5,93E-02	DDX58, IFIH1, NPM1, PRKRA, STAT2, TP53, TRAF3 (7)
Activation of IRF by Cytosolic Pattern Recognition Receptors	7,06E00	7,94E-02	DDX58, IFIH1, PPIB, STAT2, TRAF3 (5)
Cell Cycle: G1/S Checkpoint Regulation	6,92E00	7,46E-02	CCND3, GSK3B, RB1, TGFB1, TP53 (5)
Cyclins and Cell Cycle Regulation	6,51E00	6,17E-02	CCND3, GSK3B, RB1, TGFB1, TP53 (5)
Systemic Lupus Erythematosus In B Cell Signaling Pathway	6,26E00	2,5E-02	CCND3, GSK3B, IFIH1, MTOR, STAT2, TGFB1, TRAF3 (7)
EIF2 Signaling	5,56E00	2,64E-02	EIF3E, EIF3F, EIF3I, GSK3B, PABPC1, RPL19 (6)
Autophagy	5,28E00	6,15E-02	LAMP2, MAP1LC3B, MTOR, SQSTM1 (4)
FAT10 Signaling Pathway	5,18E00	1,43E-01	MAP1LC3B, PSMD4, SQSTM1 (3)
Regulation of eIF4 and p70S6K Signaling	5,04E00	3,11E-02	EIF3E, EIF3F, EIF3I, MTOR, PABPC1 (5)
Role of p14/p19ARF in Tumor Suppression	4,74E00	1,03E-01	NPM1, RB1, TP53 (3)

* number of molecules in the pathway present in the input divided by the total number of proteins in that pathway.

### Immune system pathways

SARS-CoV infects poorly monocytes/macrophages, although viral proteins are expressed, replication is incomplete in these cell types, which in turn respond secreting low levels of IFN-β and high levels of chemokines like IP-10 and MCP-1 and may be part of the inflammatory response that participates in the pathogenesis of the disease (Cheung *et al.*, 2005); dendritic cells infected by SARS-CoV induce low levels of IFN-α, IFN- β, IFN-γ, IL12p40, moderate levels of TNF- α and IL-6 and high levels of MIP-1A, IP-10, and MCP-1 (Law *et al.*, 2005). SARS-CoV shows a delayed induction of IFN- α (Spiegel, 2006). A comparison of SARS-CoV to Vesicular Stomatitis Virus (VSV) and Newcastle virus indicates that SARS-CoV induces lower levels of IFN-α, β, and γ, regardless of viral replication. Unlike SARS-CoV, MERS-CoV is able to establish an infection in human macrophages and induce higher levels of IL-12, IFN- γ, IP-10, MCP-1, MIP-1A, RANTES, and IL-8 (Zhou *et al.*, 2014).

The low activation of the IFN pathway is mediated by viral regulation of IRF3, a transcription factor activated by phosphorylation or polyubiquitination, and then translocates to the nucleus and induces the IFN response genes (Chattopadhyay *et al.*, 2016). The SARS-CoV PL^pro^ inhibits IRF3 phosphorylation, preventing its nuclear translocation and disrupting the IFN response, probably through inhibition of STING (stimulator of interferon genes), which is responsible for IRF3 phosphorylation (Chen *et al.*, 2014). Nsp3 DUB domain of MHV-A59 and SARS-CoV promotes deubiquitination of IRF3 and also prevents its activation, blocking NF-κB signaling (Frieman *et al.*, 2009). PL^pro^ of MERS-CoV has also been described to inhibit IRF3 nuclear translocation (Yang *et al.*, 2014). Interferon inhibition makes PL^pro^ an important determinator of virulence of coronavirus (Niemeyer *et al.*, 2018). The SARS-CoV Nsp1 protein also participates in IFN inhibition through decreasing STAT1 phosphorylation (Wathelet *et al.*, 2007). While the ORF3a protein induces ER stress, activates PERK (PKR-like ER Kinase), and promotes phosphorylation, ubiquitination, and degradation of IFNAR1, attenuating interferon response (Minakshi *et al.*, 2009). The SARS-CoV N protein has also been described to inhibit IFN production at an early stage, by sequestering PACT (protein activator of the dsRNA activated protein kinase R) and TRIM25 (tripartite motif protein 25), which bind to RIG-I (retinoic acid-inducer gene I) and MDA5 (melanoma differentiation gene 5) and activate IFN production (Ding *et al.*, 2017). Finally, ORF4b of MERS-CoV is another protein that has been characterized to inhibit IFN and NF-κB signaling (Matthews *et al.*, 2014).

STAT3 modulation plays an important role in pro- and anti-inflammatory responses. As already mentioned, SARS-CoV PL^pro^ activates TGF-β1 through the p38MAPK/ERK1-2 pathway, promoting STAT3 activation (Li *et al.*, 2016). MERS-CoV strains with mutations in the NSP3 and ORF4a display differential STAT3 activation and different inflammatory cytokine profiles (Selinger *et al.*, 2014). In SARS-CoV infection, a reduction of IL-4 is observed, which participates in humoral protection, an increase of IFN- γ, that participates in a potent cell-mediated immune response and also the elevation of IL-10 that plays a part in disease susceptibility (Zhu, 2004).

### Cell cycle pathways

SARS-CoV has also been described to arrest the cell cycle. Nucleocapsid protein was shown to arrest cell cycle at the S phase, through direct interaction with cyclin D and inhibition of the CDK4/Cyclin D complex, preventing phosphorylation of Rb (Retinoblastoma) protein, a central player in cell cycle control (Surjit *et al*., 2006). IBV infection also reduces Cyclin D1, which participates in G2/M transition, inducing cell cycle arrest at G2/M (Harrison *et al.*, 2007).

The blockage of G0/G1 progression has been observed by SARS-CoV ORF7a and ORF3a proteins through the reduction of cyclin D3 expression, decreased activity of cyclin D/CDK4/6, and inhibition of Rb phosphorylation (Yuan *et al.*, 2006). Nsp15 is able to alter cellular localization of Rb and function, promoting pRb ubiquitination and degradation, increasing the proportion of S-phase cells, while overexpression of ORF4 (3b) protein arrests cell cycle at G0/G1 and promotes apoptosis (Yuan *et al.*, 2005; Bhardwaj *et al.*, 2012).

Expression of viral proteins regulates cell fate, not only cell cycle, but also controls apoptosis given its importance for viral replication. The SARS-CoV S protein suppresses the extrinsic apoptotic pathway, downregulating TRAIL and FasL, and activates the intrinsic apoptotic pathway through upregulation of Bax and down-regulation of Bcl-2, Mcl-1, Bcl-xL, and MDM2, leading to increased levels of p53 and p21 induction and G1/S arrest (Yeung *et al.*, 2008). The ORF9b protein, when accumulated in the nucleus, induces caspase 3-mediated apoptosis (Sharma *et al*., 2011). Inhibition of apoptosis is also mediated by the SARS-CoV E protein, which down-regulates IRE-1 (inositol-requiring enzyme-1) and DUSP1/10 proteins, critical regulators of innate immune response and apoptosis (DeDiego *et al.*, 2011). The SUD domain of PL^pro^ interacts with RCHY1 and promotes p53 degradation, playing a role in cell cycle and apoptosis control, whereas p53 overexpression was able to inhibit viral replication (Ma-Lauer *et al.*, 2016). SARS-CoV promotes the expression of a truncated form of p53 that inhibits apoptosis mediated by wild-type p53 (Leong *et al.*, 2005). This is supported by the observation that the Porcine epidemic diarrhea virus (PEDV) production is increased in p53 knockout cells (Hao *et al.*, 2019).

### Protein synthesis control pathways

Protein synthesis pathways are often modulated by viruses. The activation of the PKR pathway by RNA viruses is an important cellular defense mechanism, which is in several cases counteracted by viruses, including coronaviruses. For example, the Dengue virus sustains, at early stages of infection, activation of the cap-dependent machinery, switching the protein synthesis to a cap-independent process in the late stages by downregulation of p70-S6K, 4E-BP1 and eIF4 factors (Villas-Bôas *et al.*, 2009). The SARS-CoV ORF3a is known to cause endoplasmic reticulum stress and activation of eIF2α (eukaryotic initiation factor 2 alpha) and PERK, affecting innate immunity by suppression of type 1 IFN signaling (Minakshi *et al.*, 2009). PKR and PERK, which promote phosphorylation of eIF2α that may suppress host translation, are expressed at high levels during SARS-CoV replication, although knockdown of PKR does not affect viral replication (Krähling *et al.*, 2009). This suggests that SARS-CoV presents a mechanism to overcome the inhibitory effects of phosphorylated eIF2α on viral mRNA translation. On the other hand, another study has shown that depletion of the antiviral PKR pathway enhanced virus replication, increasing SARS-CoV protein expression and virus production (de Wilde *et al.*, 2015).

Metformin and rapamycin are known modulators of viral infection and translation control pathways, such as mTOR. It is reported, for example, that in the 1971 Influenza outbreak, diabetic patients treated with phenformin and buformin presented a lower incidence of infection compared to diabetics treated with sulfonylureas or insulin (Lehrer, 2020). The immunoregulation of COVID-19 with mTOR inhibitors, such as rapamycin, has been proposed recently (Zheng *et al.*, 2020). Also, since it seems that all coronaviruses rely on cap-dependent translation to produce their proteins, key eIF cap-binding complex constituents are candidates for therapeutic intervention against coronavirus diseases (Gordon *et al.*, 2020).

## Pharmacological interventions for the treatment of diseases associated with coronaviruses

Severe coronavirus infection leads to epithelial cell proliferation, macrophage infiltration in the lung (Nicholls *et al.*, 2003) and can cause pulmonary fibrosis, which can linger in recovered patients (Antonio *et al.*, 2003). About 15% of COVID-19 patients progress to acute respiratory distress syndrome (ARDS), the most severe cases should be treated in intensive care units (ICU) with oxygen therapy and mechanical ventilation (Li *et al.*, 2020). In extreme cases of COVID-19, lung transplantation is possible, if viable, as a last resort (Chen J-Y *et al.*, 2020). The pharmacological interventions against coronaviruses, as summarized in Figure 1 and Table 4, are reviewed regarding their molecular mechanisms of action, *in vitro* and *in vivo* effectiveness, and ongoing clinical trials. There are two important aspects in the clinical outcome of COVID-19: one is viral entry/replication and the second is host response. Both are intimately linked and can be targeted by different compounds.

**Table 4:** Pharmacological interventions targeting the replication cycle of human coronaviruses.

Pharmacological interventions	Targeting mechanism	Reference
Human recombinant ACE2	Virus entry: inhibition of virus binding	(Monteil *et al.*, 2020)
Arbidol	Virus entry: envelope fusion and endocytosis blockage	(Teissier *et al.*, 2011; Blaising *et al.*, 2013)
Remdesivir	Replication: adenosine analog	(Sheahan *et al.*, 2017; Brown *et al.*, 2019; Wang M *et al.*, 2020)
Ribavirin, Sofosbuvir, Galidesivir, Tenofovir Favipiravir	Replication: nucleotides analogs	(Elfiky, 2020; Cai *et al.*, 2020)
Lopinavir and Ritonavir	Protease inhibitors	(Chu *et al.*, 2004; Nutho *et al.*, 2020; Choy *et al.*, 2020)
Chloroquine, Hydroxychloroquine	Virus entry: alkalinization of acid vesicles, inhibition of virus binding	(Simmons *et al.*, 2004; Rolain *et al.*, 2007)

### Interventions on viral entry/replication

Strategies to hinder viral binding have been investigated. Human recombinant ACE2 reduced SARS-CoV-2 recovery *in vitro* and protected mice from acute lung injury caused by SARS-CoV (Monteil *et al.*, 2020). A chimeric protein composed of the extracellular domain of ACE2 fused with the Fc region of IgG1 exhibited pharmacological properties in mice (Lei *et al.*, 2020). SARS-CoV-2 has two possible entry mechanisms: through endosome or membrane fusion. Arbidol (Umifenovir) is a potent broad-spectrum antiviral that blocks viral envelope fusion (Teissier *et al.*, 2011) and clathrin-mediated endocytosis (Blaising *et al.*, 2013), suppressing the replication of SARS-CoV *in vitro* (Khamitov *et al.*, 2008). Patients treated with Arbidol had a shorter period of SARS-CoV-2 infection compared to patients treated with Lopinavir/Ritonavir (Zhu *et al.*, 2020).

Several drugs have recently been used to inhibit SARS-CoV-2 replication, including the adenosine analog Remdesivir, which targets the RNA-dependent RNA polymerase and is incorporated into viral RNA chains, resulting in premature termination. Remdesivir was firstl shown to be effective against the Ebola virus (Warren *et al.*, 2016) but presents activity against other viruses, including members of the Filoviridae, Paramyxoviridae, Pneumoviridae, and Orthocoronavirinae families (Brown *et al.*, 2019). Remdesivir inhibits SARS-CoV and MERS-CoV (Sheahan *et al.*, 2017), even though the identity among the coronavirus RdRps range from 70-90%, Remdesivir shows a broad spectrum of activity (Brown *et al.*, 2019). Recently, a study showed that Remdesivir also acts against SARS-CoV-2, according to its potential antiviral mechanism as a nucleotide analog (Wang M *et al,*, 2020). Other nucleotide analogs, such as Ribavirin, Sofosbuvir, Galidesivir, and Tenofovir, can bind to SARS-CoV-2 RdRp (Elfiky, 2020). Favipiravir, a purine analog used against Influenza, is being tested against COVID-19 and reduced the time for viral clearance compared to patients treated with Lopinavir/Ritonavir (Cai *et al.,* 2020). There are currently ongoing clinical trials to evaluate Remdesivir and Ribavirin against COVID-19 and, regardless of its cost and administration routes, there are also concerns regarding its side effects and efficacy (Khalili *et al.*, 2020).

Furthermore, studies reported that the protease inhibitors Lopinavir and Ritonavir, used as HIV antivirals, also appear to have effects against SARS-CoV and SARS-CoV-2 (Chu *et al.*, 2004; Choy *et al.*, 2020). Both drugs can interact with the protease 3CL^pro^; Ritonavir has a higher binding affinity compared with Lopinavir (Nutho *et al.*, 2020). Animal experiments against SARS-CoV and MERS-CoV showed that the combination of Lopinavir/Ritonavir (LPV/r) with IFN-β significantly reduced viral load and improved pulmonary function. The combination of LPV/r shows a synergistic effect in the treatment of SARS patients (Yao *et al.*, 2020). A clinical trial (NCT02845843) is currently testing a combination of Lopinavir, Ritonavir, and interferon-β1b against MERS (Arabi *et al.*, 2020). Combined LPV/r reduced the time for patients to test negative for SARS-CoV-2 (Yao *et al.*, 2020), and increased eosinophils, indicating an improvement in COVID-19 clinical outcome (Liu *et al.*, 2020). Another study, however, reported no difference in the administration of Lopinavir and Ritonavir in a group of patients with COVID-19 already in advanced stages (Cao *et al.*, 2020), or shortening of the duration of SARS-CoV-2 shedding (Cheng C-Y *et al.*, 2020). A retrospective analysis of adverse drug reactions (ADRs) from patients with COVID-19 admitted at the First Hospital of Changsha in China revealed that about 64% of the observed ADRs were correlated with the use of LPV/r (Sun *et al.*, 2020). Patients treated with LPV/r presented a significantly higher proportion of abnormal liver function (Fan *et al.*, 2020). There are more than 280 clinical trials ongoing, considering these antivirals, using Lopinavir, Ritonavir, Remdesivir, Favipiravir, in combination, alone or with other drugs in SARS-CoV-2 patients (Cochrane COVID-19 Study register).

Chloroquine (CQ) has also been tested against SARS-CoV. It promotes alkalinization of acid vesicles in cells infected by intracellular pathogens (Rolain *et al.*, 2007) and emerged as a substitute to quinine against malaria. CQ and hydroxychloroquine (HCQ) have been tested against viral hepatitis (Pareja-Coronel, 1963), Dengue virus (Farias *et al.*, 2015), HIV (Paton *et al.*, 2002), and also against other pathogens, such as intracellular bacteria (*Coxiela burnetii* and *Tropheryma whipplei*), bacteria-like *Legionella pneumophila* and *Mycobacterium spp*, and fungal infections by *Histoplasma capsulate* and *Cryptococcus neoformans* (Rolain *et al.*, 2007). CQ is active in Vero E6 and Huh7 cells infected with MERS-CoV (de Wilde *et al.*, 2014), but not in dendritic cells and monocyte-derived macrophages (Cong *et al.*, 2018). CQ is also active *in vitro* against SARS-CoV either before or after virus exposure, interfering with ACE2 glycosylation and inhibiting viral binding (Keyaerts *et al.*, 2004; Vincent *et al.*, 2005). In addition, CQ induces alteration of endosomal pH that inhibits viral infection (Simmons *et al.*, 2004).

Moreover, CQ and HCQ have shown some *in vivo* effects against SARS-CoV-2 (Wang M *et al.,* 2020). A group of Chinese researchers recently reported beneficial effects of chloroquine in the treatment of COVID-19, however, without yet publishing data (Gao *et al.*, 2020). Another group of French researchers reported that HCQ decreased SARS-CoV-2 levels in a small group of tested patients, and the administration of azithromycin appears to improve such effects (Gautret *et al.*, 2020). A recent review analyzed several ongoing clinical trials and indicates there are paradoxical results, some have shown beneficial results, others point to the toxicity issues (Sharma, 2020). One important point is that there are different strains of SARS-CoV-2 circulating (Wang C *et al.,* 2020). Notedly, CQ has pro-apoptotic activity and the prophylactic use of CQ has been linked to the selection of intracellular pathogen strains that promote cell resistance to apoptosis and enhanced lethality, as observed for HIV and SARS-CoV (Parris, 2004). Despite being a low-cost drug, there is a consensus among health agencies, such as the WHO, that further studies are needed for the clinical use of CQ and HCQ for COVID-19 treatment. Until now, 593 clinical trials are registered using HCQ or CQ to enlighten their role in SARS-CoV-2 infection treatment (Cochrane COVID-19 Study register).

### Interventions on host cell response

The host response to viral infection is another important factor in COVID-19. SARS-CoV-2 induces secretion of IFN-γ, IL-1β, IL-4, IL-10, IP-10, and MCP-1 (Huang *et al.*, 2020). Patients in intensive care units show higher levels of IL-2, IL-7, GCSF, IP-10, MCP-1, MIP-1A, and TNF-α that may induce cytokine storm and exacerbated inflammatory response (Huang *et al.*, 2020). SARS-CoV not only infects alveolar epithelial cells, but also vascular endothelial cells, macrophages, monocytes, and lymphocytes. Rapid viral replication causes endothelial cell damage and vascular leakage, leading to the release of pro-inflammatory cytokines. Seroconversion of the host leads to the presence of IgG anti-S protein, which may promote the accumulation of proinflammatory monocyte/macrophage and release of MCP-1 and IL-8 and have been linked to severe lung injury (Fu *et al.*, 2020). Viral clearance depends on the activation of both innate and adaptive immune responses. IFN-γ and IL-6 contribute to neutrophil recruitment and transition to the adaptive response. However, exacerbated levels of IL-6 and reduced expression of IFN-γ may decrease CD4^+^, CD8^+^, and NK cells and may be connected to cytokine storm (Lagunas-Rangel and Chávez-Valencia, 2020).

Tocilizumab is a humanized anti-IL6R monoclonal antibody that prevents IL-6 signaling. Preprint studies indicate that it is safe and shows good efficiency against COVID-19. As there is a need for more clinical trial data, its use is only suggested for critically ill patients with high levels of IL-6 (Zhang C *et al.*, 2020). Early clinical data recommends the use of repeated doses (Luo *et al.*, 2020). Some of the concerns that have been raised are about the development of osteonecrosis of the jaws and the development of acute hypertriglyceridemia (Bennardo *et al.*, 2020; Morrison *et al.*, 2020).

Interferon release is one of the most important natural defense mechanisms against viral infection. *In vivo* experiments showed that treatment with IFN-β1b reduced pulmonary infiltrates, bronchointerstitial pneumonia, and viral load against MERS-CoV (Chan *et al.*, 2015). IFN-α, a mismatched double-stranded RNA Interferon inducer, and the IFN inducer Ampligen, inhibited SARS-CoV replication in the lungs (Barnard *et al*., 2006). IFN-λ also showed activity against SARS-CoV and MERS-CoV, establishing an antiviral state and presenting minimal systemic inflammation (Prokunina-Olsson *et al.*, 2020). Antibodies against cytokines and other proteins are presented in 196 studies, while 58 focus on IFN, either by inhibiting them or by giving their recombinant form to treat patients (Cochrane COVID-19 Study register).

CQ and HCQ have also shown anti-inflammatory activity and have been used in inflammatory diseases such as rheumatoid arthritis and osteoarthritis (Sharma, 2020). CQ and HCQ intervene with lysosomal acidification, inhibiting antigen presentations, phospholipase A2, Toll-Like Receptors (TLRs), T and B cell receptors, and production of cytokines, like IL-1 and IL-6 (Sinha and Balayla, 2020). The inhibition of GSK3β by CQ may also be responsible for its immunomodulatory activity against COVID-19 (Embi *et al.,* 2020).

Metronidazole is a redox-active prodrug that reduces the levels of pro-inflammatory cytokines, increases circulating lymphocytes, and decreases ROS produced by neutrophils and has also been suggested for the treatment of COVID-19 (Gharebaghi *et al.*, 2020). Another class of anti-inflammatory drugs is the statins and these have been included in some treatment protocols. However, as statins also modulate TLR response, the use of statins in animal experiments against SARS-CoV and MERS-CoV resulted in increased viral load, severe lung damage, and death (Dashti-Khavidaki and Khalili, 2020). Nitazoxanide is used against protozoan and helminthic infection. Tizoxanide, the active form of nitazoxanide, inhibits 16 strains of Influenza A and one strain of Influenza B, Rotavirus, HCV, Yellow Fever virus, HBV, HIV, Norovirus, and others (Rossignol, 2014). Nitazoxanide reduces the viral load from different coronaviruses (Cao *et al.*, 2015) and suppresses IL-6 production in mice (Hong *et al.*, 2012), and it is also suggested for COVID-19 treatment.

In viral RNA infections, the use of nutraceuticals has been suggested to inhibit NOX2, which in turn, restores TLR7 response to single-stranded viral RNA infection and induces IFN; nutraceuticals could also up-regulate mitochondrial antiviral-signaling proteins (MAVS) and reduce pro-inflammatory signaling (McCarty and DiNicolantonio, 2020). Other than nutraceuticals, vitamins A and D, selenium, zinc, and probiotics may be beneficial for COVID-19 patients, by enhancing immunity and preventing respiratory infections (Grant *et al.*, 2020; Jayawardena *et al.*, 2020). Thus, the nutritional status of COVID-19 patients may be of further interest in future therapies since it might have an impact on the development of the disease.

The clinical progression of COVID-19 indicates that the initial symptoms are due to increased viral load and, in the following weeks of infection, seroconversion of IgG reduces viral load while some patients present worsening symptoms related to immunopathological damage (Peiris *et al.*, 2003). Convalescent plasma (CP) has been used for SARS, MERS, Ebola virus and Chikungunya virus to improve survival rate (Alzoughool and Alanagreh, 2020). Different groups have tested critically ill COVID-19 patients with CP and obtained good recovery with no severe adverse effects (Duan *et al.*, 2020; Shi *et al.*, 2020; Zhang L *et al.*, 2020). The FDA has approved CP to treat critical patients (Tanne, 2020), although it has the risk of aggravating hyperimmune response, presenting a better response if administered in the early onset of the disease (Zhao and He, 2020). Key points to the use of CP are: establishing eligibility criteria of donor COVID-19 convalescent patients, pre-screening tests of the donors, criteria for CP collection, and treatment of plasma (Epstein and Burnouf, 2020). Currently, CP is being tested in 388 clinical trials worldwide against COVID-19 (Cochrane COVID-19 Study register).

## Conclusions

In summary, the cellular mechanisms associated with coronavirus replication form a complex and integrated network of molecular events, starting from the translation of nsps, proteolytic cleavage of polyproteins, assemble of RTC, transcription of antigenome, genome and subgenomic RNAs, translation of structural proteins, and finally assembly and budding of viral particles. The analysis of the cellular proteins related to coronavirus proteins reveals the modulation of key cellular pathways related to innate immunity, cell cycle, and protein synthesis. The current therapeutic approaches for COVID19 are partially related to these molecular events and pathways, but future pharmacological interventions may benefit from a better understanding regarding the replication cycle of SARS-CoV-2.

## Supplementary material

The following online material is available for this article:

Figure S1 - Role of PKR in interferon induction and antiviral response.

Figure S2 - Activation of IRF by cytosolic pattern recognition receptors.

Figure S3 - Cell cycle: G1/S checkpoint regulation.

Figure S4 - Cyclins and cell cycle regulation.

Figure S5 - Systemic lupus erythematosus in B cell signaling pathway.

Figure S6 - EIF2 signaling.

Figure S7 - Autophagy.

Figure S8 - FAT10 signaling pathway.

Figure S9 - Regulation of eIF4 and p70S6K signaling.

Figure S10 - Role of p14/p19ARF in tumor suppression.

## References

[B1] Alzoughool F, Alanagreh L (2020). Coronavirus drugs: Using plasma from recovered patients as a treatment for COVID-19. Int J Risk Saf Med.

[B2] Angelini MM, Akhlaghpour M, Neuman BW, Buchmeier MJ (2013). Severe acute respiratory syndrome coronavirus nonstructural proteins 3, 4, and 6 induce double-membrane vesicles. MBio.

[B3] Antonio GE, Wong KT, Hui DSC, Wu A, Lee N, Yuen EHY, Leung CB, Rainer TH, Cameron P, Chung SSC (2003). Thin-section CT in patients with severe acute respiratory syndrome following hospital discharge: Preliminary experience. Radiology.

[B4] Arabi YM, Asiri AY, Assiri AM, Aziz Jokhdar HA, Alothman A, Balkhy HH, AlJohani S, Al Harbi S, Kojan S, Al Jeraisy M (2020). Treatment of Middle East respiratory syndrome with a combination of lopinavir/ritonavir and interferon-β1b (MIRACLE trial): Statistical analysis plan for a recursive two-stage group sequential randomized controlled trial. Trials.

[B5] Báez-Santos YM, St. John SE, Mesecar AD (2015). The SARS-coronavirus papain-like protease: Structure, function and inhibition by designed antiviral compounds. Antiviral Res.

[B6] Baltimore D (1971). Expression of animal virus genomes. Bacteriol Rev.

[B7] Barnard DL, Day CW, Bailey K, Heiner M, Montgomery R, Lauridsen L, Chan PKS, Sidwell RW (2006). Evaluation of immunomodulators, interferons and known in vitro SARS-coV inhibitors for inhibition of SARS-coV replication in BALB/c mice. Antivir Chem Chemother.

[B8] Beniac DR, Andonov A, Grudeski E, Booth TF (2006). Architecture of the SARS coronavirus prefusion spike. Nat Struct Mol Biol.

[B9] Bennardo F, Buffone C, Giudice A (2020). New therapeutic opportunities for COVID-19 patients with Tocilizumab: Possible correlation of interleukin-6 receptor inhibitors with osteonecrosis of the jaws. Oral Oncol.

[B10] Bhardwaj K, Sun J, Holzenburg A, Guarino LA, Kao CC (2006). RNA recognition and cleavage by the SARS coronavirus endoribonuclease. J Mol Biol.

[B11] Bhardwaj K, Liu P, Leibowitz JL, Kao CC (2012). The coronavirus endoribonuclease Nsp15 interacts with retinoblastoma tumor suppressor protein. J Virol.

[B12] Blaising J, Lévy PL, Polyak SJ, Stanifer M, Boulant S, Pécheur E-I (2013). Arbidol inhibits viral entry by interfering with clathrin-dependent trafficking. Antiviral Res.

[B13] Bos EC, Luytjes W, van der Meulen HV, Koerten HK, Spaan WJ (1996). The production of recombinant infectious DI-particles of a murine coronavirus in the absence of helper virus. Virology.

[B14] Boscarino JA, Logan HL, Lacny JJ, Gallagher TM (2008). Envelope protein palmitoylations are crucial for murine coronavirus assembly. J Virol.

[B15] Bosch BJ, van der Zee R, de Haan CAM, Rottier PJM (2003). The coronavirus spike protein is a class I virus fusion protein: Structural and functional characterization of the fusion core complex. J Virol.

[B16] Bouvet M, Imbert I, Subissi L, Gluais L, Canard B, Decroly E (2012). RNA 3’-end mismatch excision by the severe acute respiratory syndrome coronavirus nonstructural protein nsp10/nsp14 exoribonuclease complex. Proc Natl Acad Sci U S A.

[B17] Brierley I, Digard P, Inglis SC (1989). Characterization of an efficient coronavirus ribosomal frameshifting signal: Requirement for an RNA pseudoknot. Cell.

[B18] Brown AJ, Won JJ, Graham RL, Dinnon KH, Sims AC, Feng JY, Cihlar T, Denison MR, Baric RS, Sheahan TP (2019). Broad spectrum antiviral remdesivir inhibits human endemic and zoonotic deltacoronaviruses with a highly divergent RNA dependent RNA polymerase. Antiviral Res.

[B19] Cai Q, Yang M, Liu D, Chen J, Shu D, Xia J, Liao X, Gu Y, Cai Q, Yang Y (2020). Experimental treatment with Favipiravir for COVID-19: An open-label control study. Engineering.

[B20] Cao J, Forrest JC, Zhang X (2015). A screen of the NIH Clinical Collection small molecule library identifies potential anti-coronavirus drugs. Antiviral Res.

[B21] Cao B, Wang Y, Wen D, Liu W, Wang J, Fan G, Ruan L, Song B, Cai Y, Wei M (2020). A trial of lopinavir-ritonavir in adults hospitalized with severe Covid-19. N Engl J Med.

[B22] Cascarina SM, Ross ED (2020). A proposed role for the SARS-CoV-2 nucleocapsid protein in the formation and regulation of biomolecular condensates. FASEB J.

[B23] Chan JF-W, Yao Y, Yeung M-L, Deng W, Bao L, Jia L, Li F, Xiao C, Gao H, Yu P (2015). Treatment with lopinavir/ritonavir or interferon-β1b improves outcome of MERS-CoV infection in a nonhuman primate model of common marmoset. J Infect Dis.

[B24] Chang C, Sue S-C, Yu T, Hsieh C-M, Tsai C-K, Chiang Y-C, Lee S, Hsiao H, Wu W-J, Chang W-L (2006). Modular organization of SARS coronavirus nucleocapsid protein. J Biomed Sci.

[B25] Chattopadhyay S, Kuzmanovic T, Zhang Y, Wetzel JL, Sen GC (2016). Ubiquitination of the transcription factor IRF-3 activates RIPA, the apoptotic pathway that protects mice from viral pathogenesis. Immunity.

[B26] Chen H, Wurm T, Britton P, Brooks G, Hiscox JA (2002). Interaction of the coronavirus nucleoprotein with nucleolar antigens and the host cell. J Virol.

[B27] Chen SC, Lo SY, Ma HC, Li HC (2009). Expression and membrane integration of SARS-CoV e protein and its interaction with M protein. Virus Genes.

[B28] Chen Y, Cai H, Pan J, Xiang N, Tien P, Ahola T, Guo D (2009). Functional screen reveals SARS coronavirus nonstructural protein nsp14 as a novel cap N7 methyltransferase. Proc Natl Acad Sci U S A.

[B29] Chen X, Yang X, Zheng Y, Yang Y, Xing Y, Chen Z (2014). SARS coronavirus papain-like protease inhibits the type I interferon signaling pathway through interaction with the STING-TRAF3-TBK1 complex. Protein Cell.

[B30] Chen J-Y, Qiao K, Liu F, Wu B, Xu X, Jiao G-Q, Lu R-G, Li H-X, Zhao J, Huang J-A (2020). Lung transplantation as therapeutic option in acute respiratory distress syndrome for coronavirus disease 2019-related pulmonary fibrosis. Chin Med J.

[B31] Chen Y, Liu Q, Guo D (2020). Emerging coronaviruses: Genome structure, replication, and pathogenesis. J Med Virol.

[B32] Cheng C-Y, Lee Y-L, Chen C-P, Lin Y-C, Liu C-E, Liao C, Cheng S-H (2020). Lopinavir/ritonavir did not shorten the duration of SARS CoV-2 shedding in patients with mild pneumonia in Taiwan. J Microbiol Immunol Infect.

[B33] Cheung CY, Poon LLM, Ng IHY, Luk W, Sia S-F, Wu MHS, Chan K-H, Yuen K-Y, Gordon S, Guan Y (2005). Cytokine responses in severe acute respiratory syndrome coronavirus-infected macrophages in vitro: Possible relevance to pathogenesis. J Virol.

[B34] Choy K-T, Wong AY-L, Kaewpreedee P, Sia SF, Chen D, Hui KPY, Chu DKW, Chan MCW, Cheung PP-H, Huang X (2020). Remdesivir, lopinavir, emetine, and homoharringtonine inhibit SARS-CoV-2 replication in vitro. Antiviral Res.

[B35] Chu CM, Cheng VCC, Hung IFN, Wong MML, Chan KH, Chan KS, Kao RYT, Poon LLM, Wong CLP, Guan Y (2004). Role of lopinavir/ritonavir in the treatment of SARS: Initial virological and clinical findings. Thorax.

[B36] Cong Y, Hart BJ, Gross R, Zhou H, Frieman M, Bollinger L, Wada J, Hensley LE, Jahrling PB, Dyall J (2018). MERS-CoV pathogenesis and antiviral efficacy of licensed drugs in human monocyte-derived antigen-presenting cells. PLoS One.

[B37] Cornillez-Ty CT, Liao L, Yates JR, Kuhn P, Buchmeier MJ (2009). Severe acute respiratory syndrome coronavirus nonstructural protein 2 interacts with a host protein complex involved in mitochondrial biogenesis and intracellular signaling. J Virol.

[B38] Cottam EM, Whelband MC, Wileman T (2014). Coronavirus NSP6 restricts autophagosome expansion. Autophagy.

[B39] Cui L, Wang H, Ji Y, Yang J, Xu S, Huang X, Wang Z, Qin L, Tien P, Zhou X (2015). The nucleocapsid protein of coronaviruses acts as a viral suppressor of rna silencing in mammalian cells. J Virol.

[B40] Dashti-Khavidaki S, Khalili H (2020). Considerations for statin therapy in patients with COVID-19. Pharmacotherapy.

[B41] de Haan CAM, Rottier PJM (2005). Molecular interactions in the assembly of coronaviruses. Adv Virus Res.

[B42] de Wilde AH, Jochmans D, Posthuma CC, Zevenhoven-Dobbe JC, van Nieuwkoop S, Bestebroer TM, van den Hoogen BG, Neyts J, Snijder EJ (2014). Screening of an FDA-approved compound library identifies four small-molecule inhibitors of Middle East respiratory syndrome coronavirus replication in cell culture. Antimicrob Agents Chemother.

[B43] de Wilde AH, Wannee KF, Scholte FEM, Goeman JJ, Ten Dijke P, Snijder EJ, Kikkert M, van Hemert MJ (2015). A kinome-wide small interfering RNA screen identifies proviral and antiviral host factors in severe acute respiratory syndrome coronavirus replication, including double-stranded RNA-activated protein kinase and early secretory pathway proteins. J Virol.

[B44] Decroly E, Debarnot C, Ferron F, Bouvet M, Coutard B, Imbert I, Gluais L, Papageorgiou N, Sharff A, Bricogne G (2011). Crystal structure and functional analysis of the SARS-coronavirus RNA cap 2’-O-methyltransferase nsp10/nsp16 complex. PLoS Pathog.

[B45] Dedeurwaerder A, Olyslaegers DAJ, Desmarets LMB, Roukaerts IDM, Theuns S, Nauwynck HJ (2014). ORF7-encoded accessory protein 7a of feline infectious peritonitis virus as a counteragent against IFN-α-induced antiviral response. J Gen Virol.

[B46] DeDiego ML, Alvarez E, Almazán F, Rejas MT, Lamirande E, Roberts A, Shieh W-J, Zaki SR, Subbarao K, Enjuanes L (2007). A severe acute respiratory syndrome coronavirus that lacks the E gene is attenuated in vitro and in vivo. J Virol.

[B47] DeDiego ML, Nieto-Torres JL, Jiménez-Guardeño JM, Regla-Nava JA, Álvarez E, Oliveros JC, Zhao J, Fett C, Perlman S, Enjuanes L (2011). Severe acute respiratory syndrome coronavirus envelope protein regulates cell stress response and apoptosis. PLoS Pathog.

[B48] Deng X, Hackbart M, Mettelman RC, O’Brien A, Mielech AM, Yi G, Kao CC, Baker SC (2017). Coronavirus nonstructural protein 15 mediates evasion of dsRNA sensors and limits apoptosis in macrophages. Proc Natl Acad Sci U S A.

[B49] Ding Z, Fang L, Yuan S, Zhao L, Wang X, Long S, Wang M, Wang D, Foda MF, Xiao S (2017). The nucleocapsid proteins of mouse hepatitis virus and severe acute respiratory syndrome coronavirus share the same IFN-β antagonizing mechanism: Attenuation of PACT-mediated RIG-I/MDA5 activation. Oncotarget.

[B50] Duan K, Liu B, Li C, Zhang H, Yu T, Qu J, Zhou M, Chen L, Meng S, Hu Y (2020). Effectiveness of convalescent plasma therapy in severe COVID-19 patients. Proc Natl Acad Sci U S A.

[B51] Eckerle LD, Becker MM, Halpin RA, Li K, Venter E, Lu X, Scherbakova S, Graham RL, Baric RS, Stockwell TB (2010). Infidelity of SARS-CoV Nsp14-exonuclease mutant virus replication is revealed by complete genome sequencing. PLoS Pathog.

[B52] Elfiky AA (2020). Ribavirin, Remdesivir, Sofosbuvir, Galidesivir, and Tenofovir against SARS-CoV-2 RNA dependent RNA polymerase (RdRp): A molecular docking study. Life Sci.

[B53] Embi MN, Ganesan N, Sidek HM (2020). Is GSK3β a molecular target of chloroquine treatment against COVID-19?. Drug Discov Ther.

[B54] Emmott E, Munday D, Bickerton E, Britton P, Rodgers MA, Whitehouse A, Zhou E-M, Hiscox JA (2013). The cellular interactome of the coronavirus infectious bronchitis virus nucleocapsid protein and functional implications for virus biology. J Virol.

[B55] Epstein J, Burnouf T (2020). Points to consider in the preparation and transfusion of COVID‐19 convalescent plasma. Vox Sang.

[B56] Fan Z, Chen L, Li J, Cheng X, Yang J, Tian C, Zhang Y, Huang S, Liu Z, Cheng J (2020). Clinical features of COVID-19-related liver functional abnormality. Clin Gastroenterol Hepatol.

[B57] Farias KJS, Machado PRL, Muniz JAPC, Imbeloni AA, da Fonseca BAL (2015). Antiviral activity of chloroquine against dengue virus type 2 replication in Aotus monkeys. Viral Immunol.

[B58] Fehr AR, Perlman S (2015). Coronaviruses: An overview of their replication and pathogenesis. Methods Mol Biol.

[B59] Frieman M, Yount B, Heise M, Kopecky-Bromberg SA, Palese P, Baric RS (2007). Severe acute respiratory syndrome coronavirus ORF6 antagonizes STAT1 function by sequestering nuclear import factors on the rough endoplasmic reticulum/Golgi membrane. J Virol.

[B60] Frieman M, Ratia K, Johnston RE, Mesecar AD, Baric RS (2009). Severe acute respiratory syndrome coronavirus papain-like protease ubiquitin-like domain and catalytic domain regulate antagonism of IRF3 and NF-κB signaling. J Virol.

[B61] Fu Y, Cheng Y, Wu Y (2020). Understanding SARS-CoV-2-mediated inflammatory responses: From mechanisms to potential therapeutic tools. Virol Sin.

[B62] Gadlage MJ, Sparks JS, Beachboard DC, Cox RG, Doyle JD, Stobart CC, Denison MR (2010). Murine hepatitis virus nonstructural protein 4 regulates virus-induced membrane modifications and replication complex function. J Virol.

[B63] Gao J, Tian Z, Yang X (2020). Breakthrough: Chloroquine phosphate has shown apparent efficacy in treatment of COVID-19 associated pneumonia in clinical studies. Biosci Trends.

[B64] Gautret P, Lagier J-C, Parola P, Hoang VT, Meddeb L, Mailhe M, Doudier B, Courjon J, Giordanengo V, Vieira VE (2020). Hydroxychloroquine and azithromycin as a treatment of COVID-19: Results of an open-label non-randomized clinical trial. Int J Antimicrob Agents.

[B65] Gharebaghi R, Heidary F, Moradi M, Parvizi M (2020). Metronidazole; a potential novel addition to the COVID-19 treatment regimen. Arch Acad Emerg Med.

[B66] Gordon DE, Jang GM, Bouhaddou M, Xu J, Obernier K, White KM, O’Meara MJ, Rezelj VV, Guo JZ, Swaney DL (2020). A SARS-CoV-2 protein interaction map reveals targets for drug repurposing. Nature.

[B67] Grant WB, Lahore H, McDonnell SL, Baggerly CA, French CB, Aliano JL, Bhattoa HP (2020). Evidence that Vitamin D supplementation could reduce risk of influenza and COVID-19 infections and deaths. Nutrients.

[B68] Hao Z, Fu F, Cao L, Guo L, Liu J, Xue M, Feng L (2019). Tumor suppressor p53 inhibits porcine epidemic diarrhea virus infection via interferon-mediated antiviral immunity. Mol Immunol.

[B69] Harrison SM, Dove BK, Rothwell L, Kaiser P, Tarpey I, Brooks G, Hiscox JA (2007). Characterisation of cyclin D1 down-regulation in coronavirus infected cells. FEBS Lett.

[B70] Hoffmann M, Kleine-Weber H, Schroeder S, Krüger N, Herrler T, Erichsen S, Schiergens TS, Herrler G, Wu N-H, Nitsche A (2020). SARS-CoV-2 cell entry depends on ACE2 and TMPRSS2 and is blocked by a clinically proven protease inhibitor. Cell.

[B71] Hong SK, Kim HJ, Song CS, Choi IS, Lee JB, Park SY (2012). Nitazoxanide suppresses IL-6 production in LPS-stimulated mouse macrophages and TG-injected mice. Int Immunopharmacol.

[B72] Huang C, Lokugamage KG, Rozovics JM, Narayanan K, Semler BL, Makino S (2011). SARS coronavirus nsp1 protein induces template-dependent endonucleolytic cleavage of mRNAs: Viral mRNAs are resistant to nsp1-induced RNA cleavage. PLoS Pathog.

[B73] Huang C, Wang Y, Li X, Ren L, Zhao J, Hu Y, Zhang L, Fan G, Xu J, Gu X (2020). Clinical features of patients infected with 2019 novel coronavirus in Wuhan, China. Lancet.

[B74] Hurst KR, Kuo L, Koetzner CA, Ye R, Hsue B, Masters PS (2005). A major determinant for membrane protein interaction localizes to the carboxy-terminal domain of the mouse coronavirus nucleocapsid protein. J Virol.

[B75] Hurst KR, Koetzner CA, Masters PS (2009). Identification of in vivo-interacting domains of the murine coronavirus nucleocapsid protein. J Virol.

[B76] Hurst KR, Koetzner CA, Masters PS (2013). Characterization of a critical interaction between the coronavirus nucleocapsid protein and nonstructural protein 3 of the viral replicase-transcriptase complex. J Virol.

[B77] Jayawardena R, Sooriyaarachchi P, Chourdakis M, Jeewandara C, Ranasinghe P (2020). Enhancing immunity in viral infections, with special emphasis on COVID-19: A review. Diabetes Metab Syndr.

[B78] Jia Z, Yan L, Ren Z, Wu L, Wang J, Guo J, Zheng L, Ming Z, Zhang L, Lou Z (2019). Delicate structural coordination of the Severe Acute Respiratory Syndrome coronavirus Nsp13 upon ATP hydrolysis. Nucleic Acids Res.

[B79] Keyaerts E, Vijgen L, Maes P, Neyts J, Van Ranst M (2004). In vitro inhibition of severe acute respiratory syndrome coronavirus by chloroquine. Biochem Biophys Res Commun.

[B80] Khalili JS, Zhu H, Mak NSA, Yan Y, Zhu Y (2020). Novel coronavirus treatment with ribavirin: Groundwork for an evaluation concerning COVID-19. J Med Virol.

[B81] Khamitov RA, Loginova SI, Shchukina VN, Borisevich SV, Maksimov VA, Shuster AM (2008). Antiviral activity of arbidol and its derivatives against the pathogen of severe acute respiratory syndrome in the cell cultures. Vopr Virusol.

[B82] Krähling V, Stein DA, Spiegel M, Weber F, Mühlberger E (2009). Severe acute respiratory syndrome coronavirus triggers apoptosis via protein kinase R but is resistant to its antiviral activity. J Virol.

[B83] Kumar P, Gunalan V, Liu B, Chow VTK, Druce J, Birch C, Catton M, Fielding BC, Tan Y-J, Lal SK (2007). The nonstructural protein 8 (nsp8) of the SARS coronavirus interacts with its ORF6 accessory protein. Virology.

[B84] Lagunas-Rangel FA, Chávez-Valencia V (2020). High IL-6/IFN-γ ratio could be associated with severe disease in COVID-19 patients. J Med Virol.

[B85] Law HKW, Cheung CY, Ng HY, Sia SF, Chan YO, Luk W, Nicholls JM, Peiris JSM, Lau YL (2005). Chemokine up-regulation in SARS-coronavirus-infected, monocyte-derived human dendritic cells. Blood.

[B86] Lau SKP, Feng Y, Chen H, Luk HKH, Yang W-H, Li KSM, Zhang Y-Z, Huang Y, Song Z-Z, Chow W-N (2015). Severe acute respiratory syndrome (SARS) coronavirus ORF8 protein is acquired from SARS-related coronavirus from greater horseshoe bats through recombination. J Virol.

[B87] Lau SKP, Luk HKH, Wong ACP, Li KSM, Zhu L, He Z, Fung J, Chan TTY, Fung KSC, Woo PCY (2020). Possible bat origin of severe acute respiratory syndrome coronavirus 2. Emerg Infect Dis.

[B88] Lehrer S (2020). Inhaled biguanides and mTOR inhibition for influenza and coronavirus. World Acad Sci J.

[B89] Lei J, Kusov Y, Hilgenfeld R (2018). Nsp3 of coronaviruses: Structures and functions of a large multi-domain protein. Antiviral Res.

[B90] Lei C, Qian K, Li T, Zhang S, Fu W, Ding M, Hu S (2020). Neutralization of SARS-CoV-2 spike pseudotyped virus by recombinant ACE2-Ig. Nat Commun. Nat Commun.

[B91] Leong WF, Tan HC, Ooi EE, Koh DR, Chow VTK (2005). Microarray and real-time RT-PCR analyses of differential human gene expression patterns induced by severe acute respiratory syndrome (SARS) coronavirus infection of Vero cells. Microbes Infect.

[B92] Li S-W, Wang C-Y, Jou Y-J, Yang T-C, Huang S-H, Wan L, Lin Y-J, Lin C-W (2016). SARS coronavirus papain-like protease induces Egr-1-dependent up-regulation of TGF-β1 via ROS/p38 MAPK/STAT3 pathway. Sci Rep.

[B93] Li L, Li R, Wu Z, Yang X, Zhao M, Liu J, Chen D (2020). Therapeutic strategies for critically ill patients with COVID-19. Ann Intensive Care.

[B94] Liu F, Xu A, Zhang Y, Xuan W, Yan T, Pan K, Yu W, Zhang J (2020). Patients of COVID-19 may benefit from sustained Lopinavir-combined regimen and the increase of Eosinophil may predict the outcome of COVID-19 progression. Int J Infect Dis.

[B95] Lu J, Lu G, Tan S, Xia J, Xiong H, Yu X, Qi Q, Yu X, Li L, Yu H (2020). A COVID-19 mRNA vaccine encoding SARS-CoV-2 virus-like particles induces a strong antiviral-like immune response in mice. Cell Res.

[B96] Lu R, Zhao X, Li J, Niu P, Yang B, Wu H, Wang W, Song H, Huang B, Zhu N (2020). Genomic characterisation and epidemiology of 2019 novel coronavirus: Implications for virus origins and receptor binding. Lancet.

[B97] Luo P, Liu Y, Qiu L, Liu X, Liu D, Li J (2020). Tocilizumab treatment in COVID-19: A single center experience. J Med Virol.

[B98] Ma-Lauer Y, Carbajo-Lozoya J, Hein MY, Müller MA, Deng W, Lei J, Meyer B, Kusov Y, Von Brunn B, Bairad DR (2016). p53 down-regulates SARS coronavirus replication and is targeted by the SARS-unique domain and PL pro via E3 ubiquitin ligase RCHY1. Proc Natl Acad Sci U S A.

[B99] Marazzi I, Ho JSY, Kim J, Manicassamy B, Dewell S, Albrecht RA, Seibert CW, Schaefer U, Jeffrey KL, Prinjha RK (2012). Suppression of the antiviral response by an influenza histone mimic. Nature.

[B100] Matthews KL, Coleman CM, van der Meer Y, Snijder EJ, Frieman MB (2014). The ORF4b-encoded accessory proteins of Middle East respiratory syndrome coronavirus and two related bat coronaviruses localize to the nucleus and inhibit innate immune signalling. J Gen Virol.

[B101] McCarty MF, DiNicolantonio JJ (2020). Nutraceuticals have potential for boosting the type 1 interferon response to RNA viruses including influenza and coronavirus. Prog Cardiovasc Dis.

[B102] Minakshi R, Padhan K, Rani M, Khan N, Ahmad F, Jameel S (2009). The SARS Coronavirus 3a protein causes endoplasmic reticulum stress and induces ligand-independent downregulation of the type 1 interferon receptor. PLoS One.

[B103] Minakshi R, Padhan K, Rehman S, Hassan MI, Ahmad F (2014). The SARS Coronavirus 3a protein binds calcium in its cytoplasmic domain. Virus Res.

[B104] Monteil V, Kwon H, Prado P, Hagelkrüys A, Wimmer RA, Stahl M, Leopoldi A, Garreta E, Hurtado Del Pozo C, Prosper F (2020). Inhibition of SARS-CoV-2 infections in engineered human tissues using clinical-grade soluble human ACE2. Cell.

[B105] Morrison AR, Johnson JM, Ramesh M, Bradley P, Jennings J, Smith ZR (2020). Acute hypertriglyceridemia in patients with COVID‐19 receiving tocilizumab. J Med Virol.

[B106] Nakagawa K, Narayanan K, Wada M, Makino S (2018). Inhibition of stress granule formation by Middle East respiratory syndrome coronavirus 4a accessory protein facilitates viral translation, leading to efficient virus replication. J Virol.

[B107] Nal B, Chan C, Kien F, Siu L, Tse J, Chu K, Kam J, Staropoli I, Crescenzo-Chaigne B, Escriou N (2005). Differential maturation and subcellular localization of severe acute respiratory syndrome coronavirus surface proteins S, M and E. J Gen Virol.

[B108] Neuman BW, Kiss G, Kunding AH, Bhella D, Baksh MF, Connelly S, Droese B, Klaus JP, Makino S, Sawicki SG (2011). A structural analysis of M protein in coronavirus assembly and morphology. J Struct Biol.

[B109] Nicholls JM, Poon LLM, Lee KC, Ng WF, Lai ST, Leung CY, Chu CM, Hui PK, Mak KL, Lim W (2003). Lung pathology of fatal severe acute respiratory syndrome. Lancet.

[B110] Niemeyer D, Mösbauer K, Klein EM, Sieberg A, Mettelman RC, Mielech AM, Dijkman R, Baker SC, Drosten C, Müller MA (2018). The papain-like protease determines a virulence trait that varies among members of the SARS-coronavirus species. PLOS Pathog.

[B111] Nieto-Torres JL, DeDiego ML, Verdiá-Báguena C, Jimenez-Guardeño JM, Regla-Nava JA, Fernandez-Delgado R, Castaño-Rodriguez C, Alcaraz A, Torres J, Aguilella VM (2014). Severe acute respiratory syndrome coronavirus envelope protein ion channel activity promotes virus fitness and pathogenesis. PLoS Pathog.

[B112] Nutho B, Mahalapbutr P, Hengphasatporn K, Pattaranggoon NC, Simanon N, Shigeta Y, Hannongbua S, Rungrotmongkol T (2020). Why are lopinavir and ritonavir effective against the newly emerged coronavirus 2019? Atomistic insights into the inhibitory mechanisms. Biochemistry.

[B113] Padhan K, Tanwar C, Hussain A, Hui PY, Lee MY, Cheung CY, Peiris JSM, Jameel S (2007). Severe acute respiratory syndrome coronavirus Orf3a protein interacts with caveolin. J Gen Virol.

[B114] Pareja-Coronel A (1963). Treatment of viral hepatitis with chloroquine. Am J Gastroenterol.

[B115] Parris GE (2004). Hypothesis links emergence of chloroquine-resistant malaria and other intracellular pathogens and suggests a new strategy for treatment of diseases caused by intracellular parasites. Med Hypotheses.

[B116] Pasternak AO, Spaan WJM, Snijder EJ (2006). Nidovirus transcription: How to make sense...?. J Gen Virol.

[B117] Paton NI, Aboulhab J, Karim F (2002). Hydroxychloroquine, hydroxycarbamide, and didanosine as economic treatment for HIV-1. Lancet.

[B118] Peiris JSM, Chu CM, Cheng VCC, Chan KS, Hung IFN, Poon LLM, Law KI, Tang BSF, Hon TYW, Chan CS (2003). Clinical progression and viral load in a community outbreak of coronavirus-associated SARS pneumonia: A prospective study. Lancet.

[B119] Pfefferle S, Schöpf J, Kögl M, Friedel CC, Müller MA, Carbajo-Lozoya J, Stellberger T, von Dall’Armi E, Herzog P, Kallies S (2011). The SARS-coronavirus-host interactome: Identification of cyclophilins as target for pan-coronavirus inhibitors. PLoS Pathog.

[B120] Prokunina-Olsson L, Alphonse N, Dickenson RE, Durbin JE, Glenn JS, Hartmann R, Kotenko SV, Lazear HM, O’Brien TR, Odendall C (2020). COVID-19 and emerging viral infections: The case for interferon lambda. J Exp Med.

[B121] Raaben M, Groot Koerkamp MJA, Rottier PJM, de Haan CAM (2007). Mouse hepatitis coronavirus replication induces host translational shutoff and mRNA decay, with concomitant formation of stress granules and processing bodies. Cell Microbiol.

[B122] Rolain J-M, Colson P, Raoult D (2007). Recycling of chloroquine and its hydroxyl analogue to face bacterial, fungal and viral infections in the 21st century. Int J Antimicrob Agents.

[B123] Rossignol J-F (2014). Nitazoxanide: A first-in-class broad-spectrum antiviral agent. Antiviral Res.

[B124] Rothan HA, Byrareddy SN (2020). The epidemiology and pathogenesis of coronavirus disease (COVID-19) outbreak. J Autoimmun.

[B125] Satterly N, Tsai P-L, van Deursen J, Nussenzveig DR, Wang Y, Faria PA, Levay A, Levy DE, Fontoura BMA (2007). Influenza virus targets the mRNA export machinery and the nuclear pore complex. Proc Natl Acad Sci U S A.

[B126] Sawicki SG, Sawicki DL, Siddell SG (2007). A contemporary view of coronavirus transcription. J Virol.

[B127] Schaecher SR, Mackenzie JM, Pekosz A (2007). The ORF7b protein of severe acute respiratory syndrome coronavirus (SARS-CoV) is expressed in virus-infected cells and incorporated into SARS-CoV particles. J Virol.

[B128] Selinger C, Tisoncik-Go J, Menachery VD, Agnihothram S, Law G, Chang J, Kelly SM, Sova P, Baric RS, Katze MG (2014). Cytokine systems approach demonstrates differences in innate and pro-inflammatory host responses between genetically distinct MERS-CoV isolates. BMC Genomics.

[B129] Sethna PB, Hofmann MA, Brian DA (1991). Minus-strand copies of replicating coronavirus mRNAs contain antileaders. J Virol.

[B130] Sharma A (2020). Chloroquine paradox may cause more damage than help fight COVID-19. Microbes Infect.

[B131] Sharma K, Åkerström S, Sharma AK, Chow VTK, Teow S, Abrenica B, Booth SA, Booth TF, Mirazimi A, Lal SK (2011). SARS-CoV 9b protein diffuses into nucleus, undergoes active Crm1 mediated nucleocytoplasmic export and triggers apoptosis when retained in the nucleus. PLoS One.

[B132] Sheahan TP, Sims AC, Graham RL, Menachery VD, Gralinski LE, Case JB, Leist SR, Pyrc K, Feng JY, Trantcheva I (2017). Broad-spectrum antiviral GS-5734 inhibits both epidemic and zoonotic coronaviruses. Sci Transl Med.

[B133] Shi P, Su Y, Li R, Liang Z, Dong S, Huang J (2019). PEDV nsp16 negatively regulates innate immunity to promote viral proliferation. Virus Res.

[B134] Shi H, Zhou C, He P, Huang S, Duan Y, Wang X, Lin K, Zhou C, Zhang X, Zha Y (2020). Successful treatment with plasma exchange followed by intravenous immunoglobulin in a critically ill patient with COVID-19. Int J Antimicrob Agents.

[B135] Simmons G, Reeves JD, Rennekamp AJ, Amberg SM, Piefer AJ, Bates P (2004). Characterization of severe acute respiratory syndrome-associated coronavirus (SARS-CoV) spike glycoprotein-mediated viral entry. Proc Natl Acad Sci U S A.

[B136] Simon-Loriere E, Holmes EC (2011). Why do RNA viruses recombine?. Nat Rev Microbiol.

[B137] Sinha N, Balayla G (2020). Hydroxychloroquine and COVID-19. Postgrad Med J.

[B138] Siu YL, Teoh KT, Lo J, Chan CM, Kien F, Escriou N, Tsao SW, Nicholls JM, Altmeyer R, Peiris JSM (2008). The M, E, and N structural proteins of the severe acute respiratory syndrome coronavirus are required for efficient assembly, trafficking, and release of virus-like particles. J Virol.

[B139] Siu K, Yuen K, Castano‐Rodriguez C, Ye Z, Yeung M, Fung S, Yuan S, Chan C, Yuen K, Enjuanes L (2019). Severe acute respiratory syndrome Coronavirus ORF3a protein activates the NLRP3 inflammasome by promoting TRAF3‐dependent ubiquitination of ASC. FASEB J.

[B140] Spiegel M (2006). Interaction of severe acute respiratory syndrome-associated coronavirus with dendritic cells. J Gen Virol.

[B141] Stobart CC, Sexton NR, Munjal H, Lu X, Molland KL, Tomar S, Mesecar AD, Denison MR (2013). Chimeric exchange of coronavirus nsp5 proteases (3CLpro) identifies common and divergent regulatory determinants of protease activity. J Virol.

[B142] Sun J, Deng X, Chen X, Huang J, Huang S, Li Y, Feng J, Liu J, He G (2020). Incidence of adverse drug reactions in COVID-19 patients in China: An active monitoring study by Hospital Pharmacovigilance System. Clin Pharmacol Ther.

[B143] Surjit M, Liu B, Chow VTK, Lal SK (2006). The nucleocapsid protein of severe acute respiratory syndrome-coronavirus inhibits the activity of cyclin-cyclin-dependent kinase complex and blocks S phase progression in mammalian cells. J Biol Chem.

[B144] Tanaka T, Kamitani W, DeDiego ML, Enjuanes L, Matsuura Y (2012). Severe acute respiratory syndrome coronavirus nsp1 facilitates efficient propagation in cells through a specific translational shutoff of host mRNA. J Virol.

[B145] Tanne JH (2020). Covid-19: FDA approves use of convalescent plasma to treat critically ill patients. BMJ.

[B146] Taylor JK, Coleman CM, Postel S, Sisk JM, Bernbaum JG, Venkataraman T, Sundberg EJ, Frieman MB (2015). Severe acute respiratory syndrome coronavirus ORF7a inhibits bone marrow stromal antigen 2 virion tethering through a novel mechanism of glycosylation interference. J Virol.

[B147] te Velthuis AJW, Arnold JJ, Cameron CE, van den Worm SHE, Snijder EJ (2010). The RNA polymerase activity of SARS-coronavirus nsp12 is primer dependent. Nucleic Acids Res.

[B148] te Velthuis AJW, van den Worm SHE, Snijder EJ (2012). The SARS-coronavirus nsp7+nsp8 complex is a unique multimeric RNA polymerase capable of both de novo initiation and primer extension. Nucleic Acids Res.

[B149] Teissier E, Zandomeneghi G, Loquet A, Lavillette D, Lavergne J-P, Montserret R, Cosset F-L, Böckmann A, Meier BH, Penin F (2011). Mechanism of inhibition of enveloped virus membrane fusion by the antiviral drug arbidol. PLoS One.

[B150] Teoh K-T, Siu Y-L, Chan W-L, Schlüter MA, Liu C-J, Peiris JSM, Bruzzone R, Margolis B, Nal B (2010). The SARS coronavirus E protein interacts with PALS1 and alters tight junction formation and epithelial morphogenesis. Mol Biol Cell.

[B151] Terada Y, Matsui N, Noguchi K, Kuwata R, Shimoda H, Soma T, Mochizuki M, Maeda K (2014). Emergence of pathogenic coronaviruses in cats by homologous recombination between feline and canine coronaviruses. PLoS One.

[B152] V’kovski P, Gerber M, Kelly J, Pfaender S, Ebert N, Braga Lagache S, Simillion C, Portmann J, Stalder H, Gaschen V (2019). Determination of host proteins composing the microenvironment of coronavirus replicase complexes by proximity-labeling. Elife.

[B153] Villas-Bôas CSA, Conceição TM, Ramírez J, Santoro ABM, Da Poian AT, Montero-Lomelí M (2009). Dengue virus-induced regulation of the host cell translational machinery. Braz J Med Biol Res.

[B154] Vincent MJ, Bergeron E, Benjannet S, Erickson BR, Rollin PE, Ksiazek TG, Seidah NG, Nichol ST (2005). Chloroquine is a potent inhibitor of SARS coronavirus infection and spread. Virol J.

[B155] Wang C, Liu Z, Chen Z, Huang X, Xu M, He T, Zhang Z (2020). The establishment of reference sequence for SARS-CoV-2 and variation analysis. J Med Virol.

[B156] Wang M, Cao R, Zhang L, Yang X, Liu J, Xu M, Shi Z, Hu Z, Zhong W, Xiao G (2020). Remdesivir and chloroquine effectively inhibit the recently emerged novel coronavirus (2019-nCoV) in vitro. Cell Res.

[B157] Warren TK, Jordan R, Lo MK, Ray AS, Mackman RL, Soloveva V, Siegel D, Perron M, Bannister R, Hui HC (2016). Therapeutic efficacy of the small molecule GS-5734 against Ebola virus in rhesus monkeys. Nature.

[B158] Wathelet MG, Orr M, Frieman MB, Baric RS (2007). Severe acute respiratory syndrome coronavirus evades antiviral signaling: Role of nsp1 and rational design of an attenuated strain. J Virol.

[B159] Yan R, Zhang Y, Li Y, Xia L, Guo Y, Zhou Q (2020). Structural basis for the recognition of SARS-CoV-2 by full-length human ACE2. Science.

[B160] Yang X, Chen X, Bian G, Tu J, Xing Y, Wang Y, Chen Z (2014). Proteolytic processing, deubiquitinase and interferon antagonist activities of Middle East respiratory syndrome coronavirus papain-like protease. J Gen Virol.

[B161] Yao T, Qian J, Zhu W, Wang Y, Wang G (2020). A systematic review of lopinavir therapy for SARS coronavirus and MERS coronavirus-A possible reference for coronavirus disease‐19 treatment option. J Med Virol.

[B162] Yeung Y-S, Yip C-W, Hon C-C, Chow KYC, Ma ICM, Zeng F, Leung FCC (2008). Transcriptional profiling of Vero E6 cells over-expressing SARS-CoV S2 subunit: Insights on viral regulation of apoptosis and proliferation. Virology.

[B163] Yuan X, Shan Y, Zhao Z, Chen J, Cong Y (2005). G0/G1 arrest and apoptosis induced by SARS-CoV 3b protein in transfected cells. Virol J.

[B164] Yuan X, Wu J, Shan Y, Yao Z, Dong B, Chen B, Zhao Z, Wang S, Chen J, Cong Y (2006). SARS coronavirus 7a protein blocks cell cycle progression at G0/G1 phase via the cyclin D3/pRb pathway. Virology.

[B165] Zeng Q, Langereis MA, van Vliet ALW, Huizinga EG, de Groot RJ (2008). Structure of coronavirus hemagglutinin-esterase offers insight into corona and influenza virus evolution. Proc Natl Acad Sci U S A.

[B166] Zeng Z, Deng F, Shi K, Ye G, Wang G, Fang L, Xiao S, Fu Z, Peng G (2018). Dimerization of coronavirus nsp9 with diverse modes enhances its nucleic acid binding affinity. J Virol.

[B167] Zhang C, Wu Z, Li J-W, Zhao H, Wang G-Q (2020). Cytokine release syndrome in severe COVID-19: Interleukin-6 receptor antagonist tocilizumab may be the key to reduce mortality. Int J Antimicrob Agents.

[B168] Zhang L, Pang R, Xue X, Bao J, Ye S, Dai Y, Zheng Y, Fu Q, Hu Z, Yi Y (2020). Anti-SARS-CoV-2 virus antibody levels in convalescent plasma of six donors who have recovered from COVID-19. Aging.

[B169] Zhang T, Wu Q, Zhang Z (2020). Probable pangolin origin of SARS-CoV-2 associated with the COVID-19 outbreak. Curr Biol.

[B170] Zhao Q, He Y (2020). Challenges of convalescent plasma therapy on COVID-19. J Clin Virol.

[B171] Zhao S, Lin Q, Ran J, Musa SS, Yang G, Wang W, Lou Y, Gao D, Yang L, He D (2020). Preliminary estimation of the basic reproduction number of novel coronavirus (2019-nCoV) in China, from 2019 to 2020: A data-driven analysis in the early phase of the outbreak. Int J Infect Dis.

[B172] Zheng Y, Li R, Liu S (2020). Immunoregulation with mTOR inhibitors to prevent COVID-19 severity: A novel intervention strategy beyond vaccines and specific antiviral medicines. J Med Virol.

[B173] Zhou J, Chu H, Li C, Wong BH-Y, Cheng Z-S, Poon VK-M, Sun T, Lau CC-Y, Wong KK-Y, Chan JY-W (2014). Active replication of Middle East respiratory syndrome coronavirus and aberrant induction of inflammatory cytokines and chemokines in human macrophages: Implications for pathogenesis. J Infect Dis.

[B174] Zhu M (2004). SARS immunity and vaccination. Cell Mol Immunol.

[B175] Zhu X, Wang D, Zhou J, Pan T, Chen J, Yang Y, Lv M, Ye X, Peng G, Fang L (2017). Porcine deltacoronavirus nsp5 antagonizes type I interferon signaling by cleaving STAT2. J Virol.

[B176] Zhu Z, Lu Z, Xu T, Chen C, Yang G, Zha T, Lu J, Xue Y (2020). Arbidol monotherapy is superior to lopinavir/ritonavir in treating COVID-19. J Infect.

[B177] Ziebuhr J, Snijder EJ, Gorbalenya AE (2000). Virus-encoded proteinases and proteolytic processing in the Nidovirales. J Gen Virol.

