# Supplementary material for: Molecular mechanisms and pharmacological interventions in the replication cycle of human coronaviruses
Source: Genet Mol Biol. 2020 Nov 23;44(1 Suppl 1):e20200212. doi: 10.1590/1678-4685-GMB-2020-0212 (PMC7731901; doi:10.1590/1678-4685-GMB-2020-0212)
Supplement: Figure S1 - [file 1415-4757-gmb-44-01-s1-e20200212-s1.pdf]

Genetics and Molecular Biology
https://doi.org/10.1590/1678-4685-GMB-2020-0212

Supplementary Material to "Molecular mechanisms and pharmacological interventions in the replication cycle of human coronaviruses"

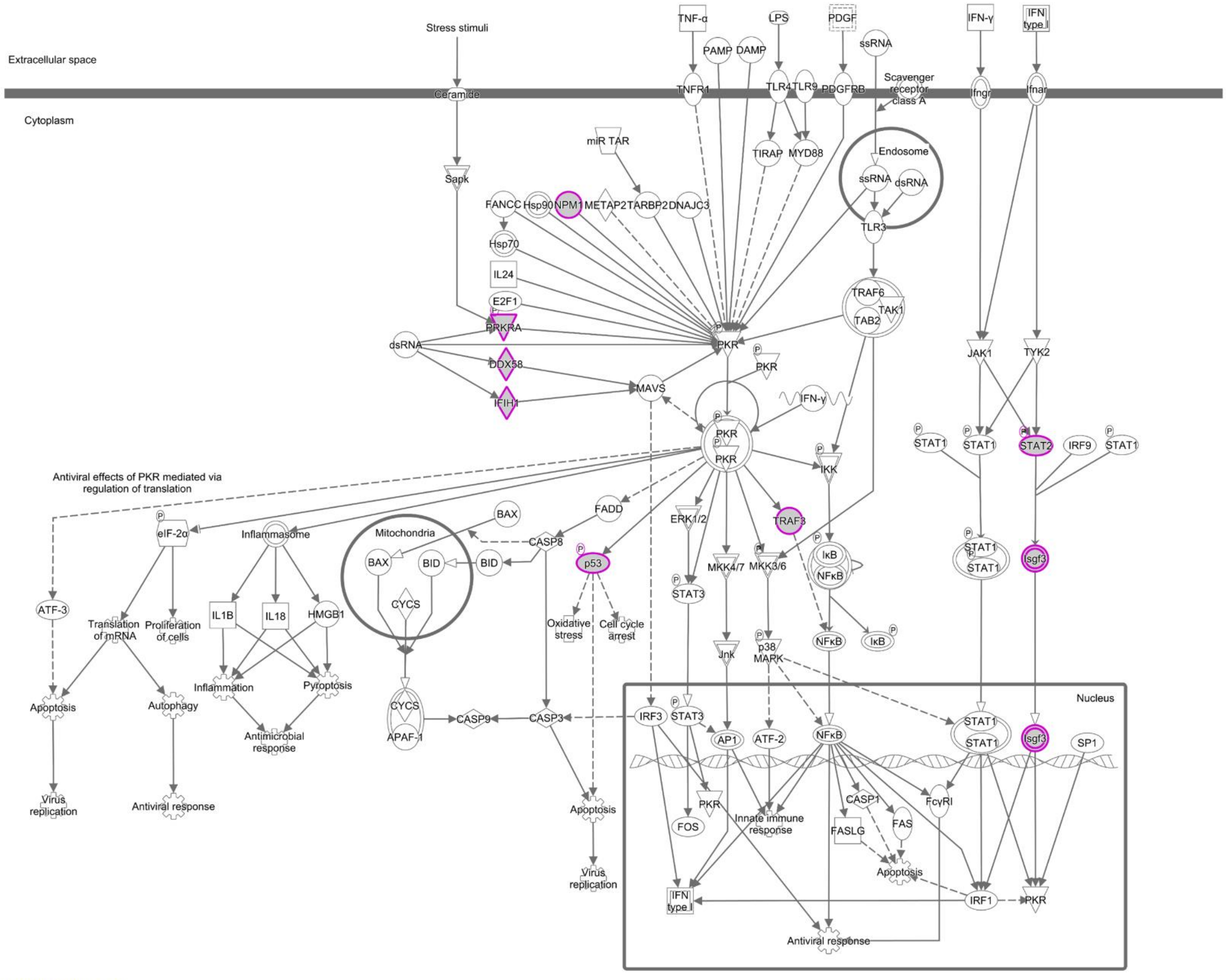

© 2000-2020 QIAGEN. All rights reserved.

Figure S1 - Role of PKR in interferon induction and antiviral response.
