# Supplementary material for: Molecular mechanisms and pharmacological interventions in the replication cycle of human coronaviruses
Source: Genet Mol Biol. 2020 Nov 23;44(1 Suppl 1):e20200212. doi: 10.1590/1678-4685-GMB-2020-0212 (PMC7731901; doi:10.1590/1678-4685-GMB-2020-0212)
Supplement: Figure S10 - [file 1415-4757-gmb-44-01-s1-e20200212-s10.pdf]

Genetics and Molecular Biology
https://doi.org/10.1590/1678-4685-GMB-2020-0212

Supplementary Material to "Molecular mechanisms and pharmacological interventions in the replication cycle of human coronaviruses"

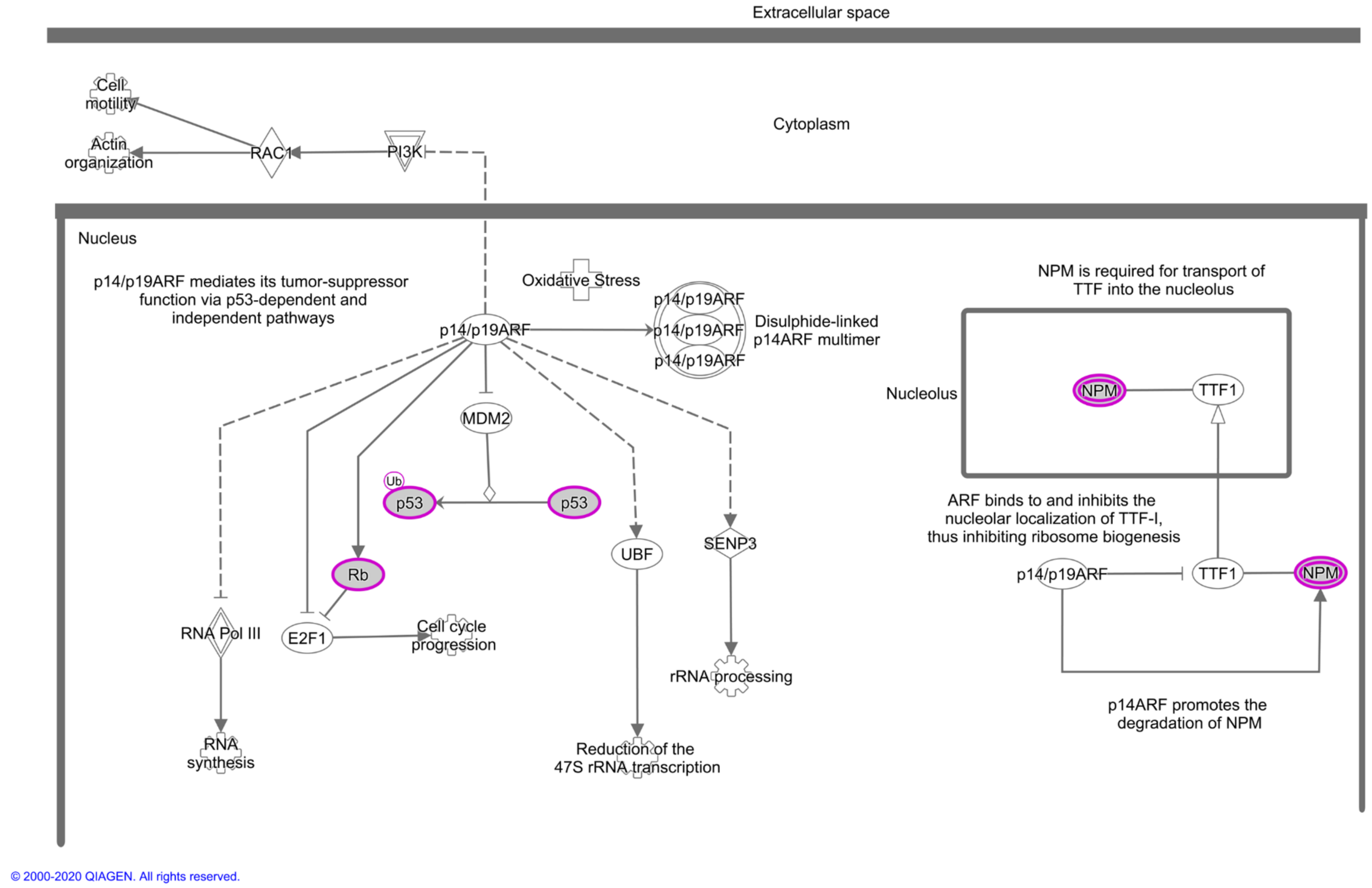

Figure S10 - Role of p14/p19ARF in tumor suppression
